# Substrate Turnover Dynamics Guide Ketol-Acid Reductoisomerase Redesign for Increased Specific Activity

**DOI:** 10.1021/acscatal.4c01446

**Published:** 2024-06-26

**Authors:** Elijah Karvelis, Chloe Swanson, Bruce Tidor

**Affiliations:** † Department of Biological Engineering, Massachusetts Institute of Technology, Cambridge, Massachusetts 02139, United States; ‡ Computer Science and Artificial Intelligence Laboratory, Massachusetts Institute of Technology, Cambridge, Massachusetts 02139, United States; § Department of Electrical Engineering and Computer Science, Massachusetts Institute of Technology, Cambridge, Massachusetts 02139, United States

**Keywords:** QM/MM molecular dynamics, path sampling, enzyme catalysis, protein design, machine learning

## Abstract

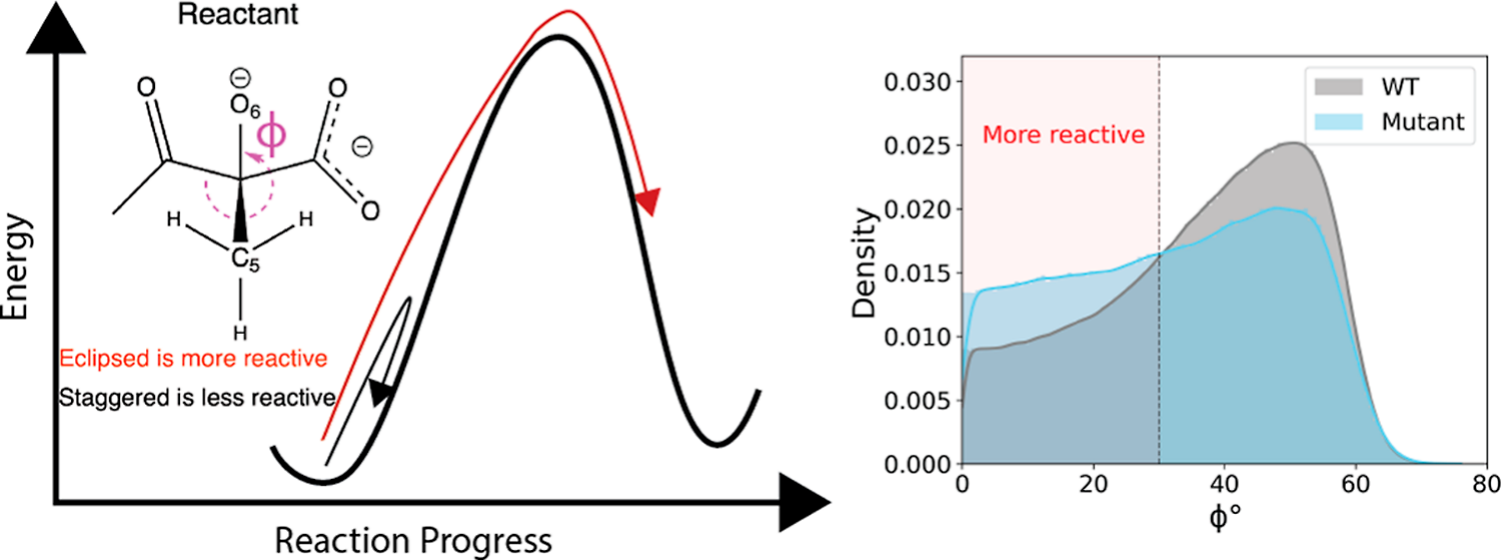

The task of adapting enzymes for specific applications is often hampered by our incomplete ability to tune and tailor catalytic functions, particularly when seeking increased activity. Here, we develop and demonstrate a rational approach to address this challenge, applied to ketol-acid reductoisomerase (KARI), which has uses in industrial-scale isobutanol production. While traditional structure-based computational enzyme redesign strategies typically focus on the enzyme-bound ground state (GS) and transition state (TS), we postulated that additionally treating the underlying dynamics of complete turnover events that connect and pass through both states could further elucidate the structural properties affecting catalysis and help identify mutations that lead to increased catalytic activity. To examine the dynamics of substrate conversion with atomistic detail, we adapted and applied computational methods based on path sampling techniques to gather thousands of QM/MM simulations of attempted substrate turnover events by KARI: both productive (reactive) and unproductive (nonreactive) attempts. From these data, machine learning models were constructed and used to identify specific conformational features (interatomic distances, angles, and torsions) associated with successful, productive catalysis. Multistate protein redesign techniques were then used to select mutations that stabilized reactive-like structures over nonreactive-like ones while also meeting additional criteria consistent with enhanced specific activity. This procedure resulted in eight high-confidence enzyme mutants with a significant improvement in calculated specific activity relative to wild type (WT), with the fastest variant’s increase in calculated *k*_cat_ being (2 ± 1) × 10^4^-fold. Collectively, these results suggest that introducing mutations designed to increase the population of reaction-promoting conformations of the enzyme–substrate complex before it reaches the barrier can provide an effective approach to engineering improved enzyme catalysts.

## Introduction

Enzymes can be highly selective and extremely efficient catalysts.^1−4^ Typically functional under mild, aqueous conditions, they are an attractive tool for sustainable industrial-scale production of pharmaceuticals, food, fuel, and commodity chemicals.^1,5−8^ Yet, despite the wide use of enzymes in hundreds of industrial processes to date, our ability to rationally tune and tailor them for enhanced activity is relatively limited, with random mutant screening and in vitro evolution typically required for successful enzyme redesign or optimization trials.^2,5,6,9,10^ The modest success of past attempts at re-engineering natural enzymes for increased activity suggests that current protein design approaches and likely our understanding of enzyme catalysis are currently insufficient for effective, rational enzyme redesign.

Redesign approaches for increasing catalytic activity typically focus on decreasing the energy of activation (Δ*G*^‡^) by stabilizing the transition state (TS) relative to the ground-state reactant (GS).^4−6,11^ This strategy is based on transition-state theory, which relates the rate of the reaction (*k*_cat_) to the height of the energy barrier between the reactants and products. While focusing on the relative energies of the GS and TS, however, these approaches typically neglect the structural and dynamic behaviors that occur in the reactant well immediately preceding the attempted reaction, as well as those occurring along the pathway connecting the GS to the TS. Nonetheless, there has been increasing evidence supporting the coupling of such dynamic behaviors to enzyme catalysis.^9,12−30^

In particular, promoting vibrations on the order of femtoseconds have been linked to turnover in purine nucleoside phosphorylase as well as artificially designed retro-aldolase enzymes.^26,28^ Conformational changes occurring on a much slower time scale have also been linked to enzyme catalysis with experimental and computational approaches, as demonstrated by Ojeda-May et al.’s study of adenylate kinase and mechanistic studies of the link between loop motions and catalysis in protein tyrosine phosphatases and TIM-barrel-containing enzymes by Kamerlin and coworkers.^18,31−34^ Conformations affecting activity have also been implicated in previous enzyme design trials. Multiple studies have characterized catalytically productive and unproductive conformational substates, the relative populations of which often tracked with overall activity in different mutants.^20,21,35^ Otten et al. used experimental techniques including nuclear magnetic resonance, crystallography, and stopped-flow experiments to explore the dynamics of a series of Kemp eliminase enzymes that were artificially evolved starting from a computational design, and they showed support for the existence of productive and unproductive substates, of which the former were increasingly sampled over the course of directed evolution.^20^ A computational study of a designed retro-aldolase that was optimized by directed evolution similarly showed evidence that progressively selected mutants increasingly stabilized catalytically efficient substates, which were defined in accordance with prior knowledge of the reaction mechanism and its relation to the arrangement of the bound substrate and surrounding catalytic residues.^21^ Population shifts away from noncatalytic conformations toward catalytic ones, resulting from experimentally selected mutations, have also been shown for the natural enzyme glucose oxidase.^35^

Given the relevance of conformational sampling for the enzyme–substrate complex to enzyme activity, there are several frameworks for studying enzyme catalysis that do not exclusively focus on the TS. These include investigations of near-attack conformations, which have suggested that lowering the barrier for the formation of subsets of GS conformations along the path toward the TS can contribute to increased activity similarly to lowering the barrier for the formation of the TS itself,^36−39^ and the computational path sampling methods,^40,41^ which are statistical mechanical techniques for directly computing the rate of a chemical reaction from ensembles of transition pathways. These path sampling approaches do not rely on transition state theory and compute reaction rates without knowledge of either the TS or a valid reaction coordinate connecting the reactant and product wells on the free energy surface.

Computational path sampling methods enable the calculation of reaction rates as well as the analysis of the unbiased dynamics underlying transitional events.^9,24,25,40−45^ Given an order parameter to distinguish between reactant and product states, which need not accurately reflect the reaction coordinate, transition path sampling (TPS) and transition interface sampling (TIS) are two techniques that can each collect attempted reactions that satisfy the requirements of Boltzmann sampling.^40,41^ Established methods analyzing the resulting ensembles of enzyme-catalyzed reactions can then uncover reaction coordinates, TSs, energies of activation, and catalytic rates,^40,41,44,46^ while further examination of the reaction trajectories can reveal structural and dynamic behaviors related to the enzyme mechanism. One particularly useful framework is to use TIS to sample both successful reactions and failed reactions (when the enzyme–substrate complex leaves the reactant well and makes progress along the reaction but does not reach the product well), which can then be compared to determine the attributes of successful versus failed catalysis at the atomic level. This approach was taken in our group’s previous study of ketol-acid reductoisomerase (KARI), which demonstrated the existence of a subregion of the enzyme–substrate complex phase space, inside the reactant well, that was associated with increased reaction efficiency.^9^ This subregion could be defined using only 10 geometric features (interatomic distances, angles, and torsions) describing the structure of the active site, and theoretical enzyme variants that passed through this reactive subregion while approaching the reaction barrier were shown to be more catalytically efficient than wild-type (WT) enzyme by many orders of magnitude.^9^ The findings reported here build on these past results and implement and test an enzyme redesign pipeline based on the notion that reactant well structural preferences can guide successful enzyme redesign for increased activity.

**Figure 1:**
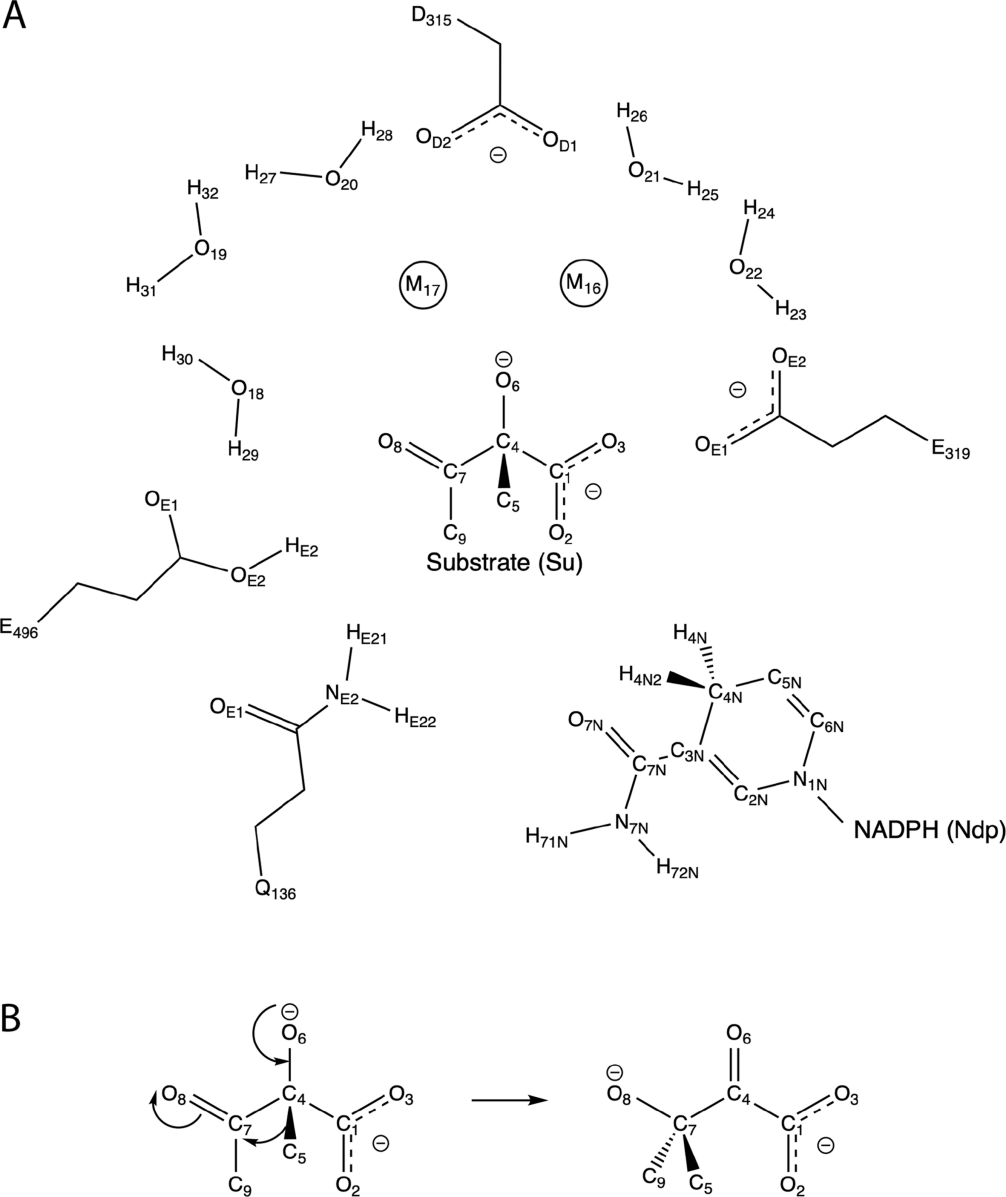
KARI active site and isomerization reaction. (A) The active site of KARI, shown here in its reactant form prior to the methyl migration reaction, binds two Mg^2+^ ion cofactors (M_16_ and M_17_), NADPH (Ndp), and substrate (Su). The active site also contains five Mg^2+^ ion-coordinating water molecules as well as the catalytic residues D315, E319, and E496. Residue Q136 interacts with NADPH. (B) Isomerization step catalyzed by KARI.

Similar to Bonk et al., we chose KARI from *Spinacia oleracea* (PDB accession number 1YVE) as our model enzyme, due to the availability of a high-quality structure and previous experimental and theoretical findings.^9,47−51^ In its active site, this homodimeric enzyme binds two divalent Mg^2+^ cations, NADPH, and one of two potential substrates: (2*S*)-acetolactate or (2*S*)-2-aceto-2-hydroxybutyrate (Figure 1A).^47−51^ Here, we focus only on its activity on (2*S*)-acetolactate, which is relevant for the industrial production of isobutanol.^52^ In the same active site, KARI catalyzes two consecutive transformations on this substrate: first, a rate-limiting isomerization reaction involving a methyl transfer (Figure 1B), and subsequently a faster NADPH-dependent reduction, ultimately producing (2*R*)-2,3-dihydroxy-3-isovalerate.^47,53−55^ For the scope of this research, we study the isomerization reaction, which, due to its role as the rate-limiting step, could be engineered to improve the prospects for large-scale isobutanol production using currently KARI-limited pathways.^47,52,53^ Relative to most natural enzymes with turnover numbers on the order of 10^4^ s^–1^ and up to 6 × 10^5^ s^–1^, KARI has a low turnover number (approximately 1 s^–1^),^48,56^ and when released from its evolutionary constraint for activity on multiple substrates, KARI’s theoretical upper limit level of activity on (2*S*)-acetolactate could be higher than its natural level of activity.

**Figure 2:**
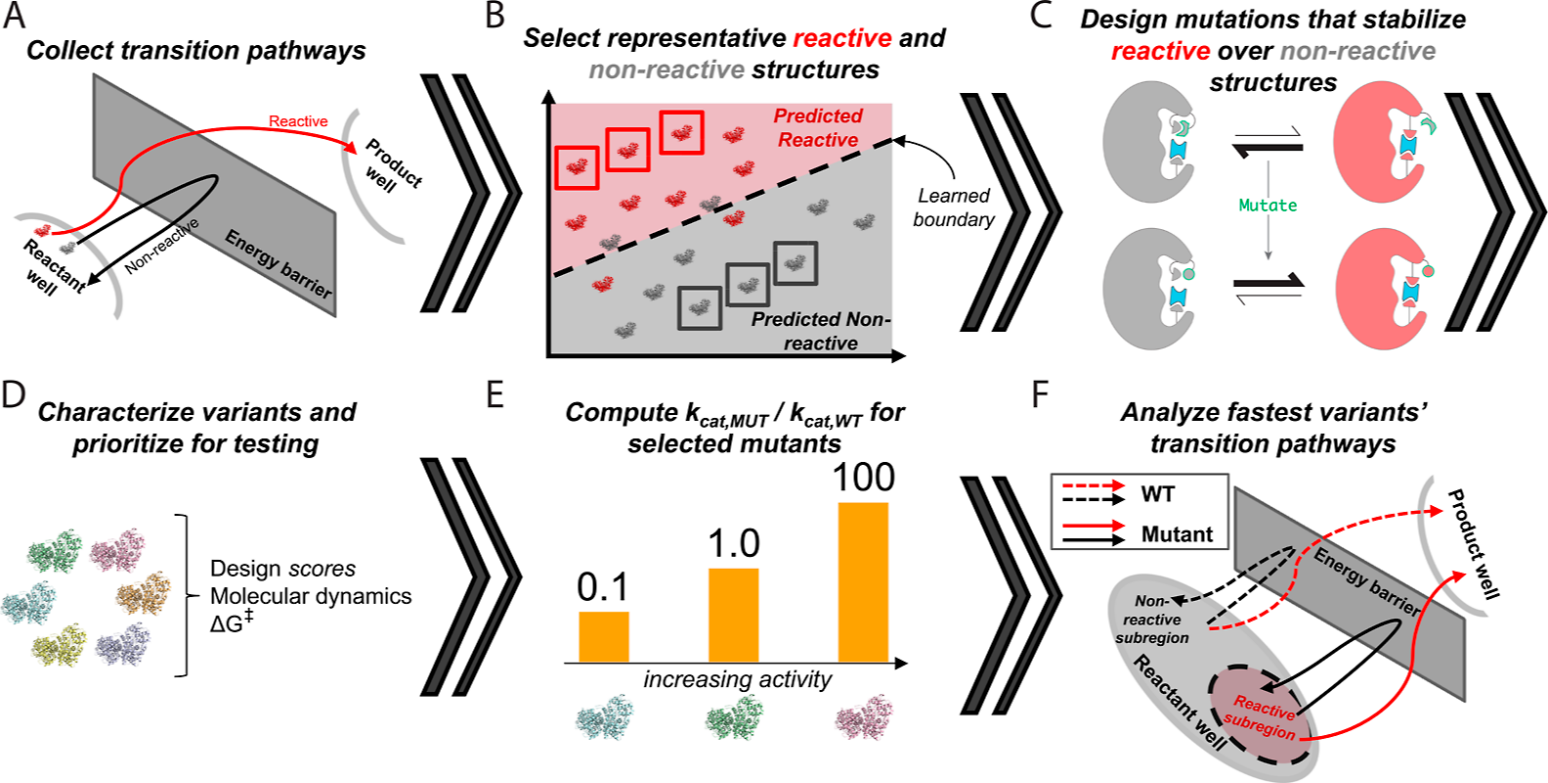
Design pipeline. (A) Ensembles of transition pathways by which the enzyme attempts to convert substrate to product were collected; by design, some attempts were successful (reactive) while others failed (nonreactive). (B) These pathways were used to learn general differences between reactant well enzyme–substrate complexes that later reach product (reactive structures) or not (nonreactive structures), and (C) mutations were selected based on their ability to stabilize reactive-like structures (red) relative to nonreactive-like structures (gray). (D) These mutants were characterized and prioritized for (E) detailed downstream TIS-based *k*_cat_ calculations. (F) Successful mutants were further characterized by comparing their transition pathways to those of WT.

A two-step approach was used in the current work: first, we established a computational enzyme redesign strategy to identify KARI mutants with increased populations of conformations corresponding to highly reactive subregions of the reactant well. Second, using simulation methodology that parallels that used to identify reactive subregions of conformation space, we evaluated whether such mutants have increased levels of overall activity relative to that of WT (Figure 2). Between these two steps, we computationally characterized candidate mutants to eliminate those with properties inconsistent with enhanced catalysis. This was important both to ensure that candidates truly did spend more time in the reactive portion of the enzyme–substrate complex well and that detrimental changes after the complex left the reactant well were not introduced.

The computational redesign approach (Figure 2) began with an atomistically detailed characterization of the reaction, as it occurred inside the WT enzyme’s active site, using QM/MM simulations of successful and failed reaction attempts sampled with TIS. These simulations were used to compute the specific rate constant *k*_cat_ and to identify and compare productive (i.e., reactive) and unproductive (i.e., nonreactive) prereaction enzyme–substrate conformations, which were respectively associated with successful and failed turnover attempts. The multistate, physics-based protein redesign algorithm COMETS^57^ then selected hundreds of mutations that stabilized productive, reactive-like conformations relative to unproductive, nonreactive-like ones. While selecting mutations to increase populations of reactive-like structures was expected to increase specific activity by way of diminishing catalytic failure modes observed for WT, introducing mutations had the potential to additionally open new failure modes, such as an increased activation energy. That is, the population of reactive-like structures was expected to be one out of several factors affecting overall activity. Therefore, different metrics and analyses, including dynamical calculations of mutant proclivities for reactive-like conformations and equilibrium calculations of approximated reaction barriers, were explored as ways to prioritize candidates for further testing. Ultimately, a subset of mutants was tested for increased activity by calculating *k*_cat_ using TIS. Over half of the tested mutants had computed *k*_cat_ larger than WT’s, among which statistical testing identified eight high-confidence mutants with significant increases in computed *k*_cat_ (the largest increase being 20,000-fold). Subsequent analysis identified structural features that could explain the increased activities of several of the fastest mutants.

Many physics-based protein engineering technologies rely on searching over a design space to optimize an objective function that directly corresponds to an engineering goal. For example, to enhance protein stability, it is typical to search over the side chain mutational space to optimize an objective function corresponding to the free energy of unfolding. Likewise, a common enzyme engineering approach to enhance specific activity is to search over side chain mutational space to optimize an objective function corresponding to the free energy of activation, which through transition state theory and together with the transmission coefficient is directly related to the rate. The design approach itself does not need to be validated because one optimizes essentially directly on the goal. Experimental testing is essential, however, to show that the work can be practically applied to real-world goals.

Here, the situation is somewhat different. The search is carried out over side chain mutational space but to optimize a function that is at best indirectly related to rate—the stabilization of a small sample of reactive-like complexes over their nonreactive-like counterparts. It is an open question whether the rigorous theoretical and computational framework applied in this type of work will evaluate design suggestions coming from this approach as successful in accelerating rate; they may all fail because, as one example, the reactive-like conformational space for each mutant may be sufficiently different from that of the WT that the objective function designed from the WT has no relevance for the mutant. Other failure modes could be that the barrier height increases in too many designs or other kinetic bottlenecks are introduced into the reaction pathway. Computationally testing the mutants against the kinetic procedure used here can be used to estimate the frequency of such failure modes and determine whether they make the proposed approach infeasible. That is, computational testing can demonstrate that the design pipeline, although manipulating reaction features only indirectly related to the rate, provides sufficient leverage to achieve enzyme engineering goals. This would be a significant result because it opens the door to a new approach toward enzyme redesign for enhanced specific activity that does not explicitly aim to lower the energy of activation, and this is the contribution of the current work. Experimental testing of these mutants, which should also be done, will tell us more about the accuracy of the energy functions and QM/MM approaches but would not specifically address the feasibility of the design hypothesis embodied in this approach.

## Results and Discussion

### Characterization of Reaction in Wild Type

**Figure 3:**
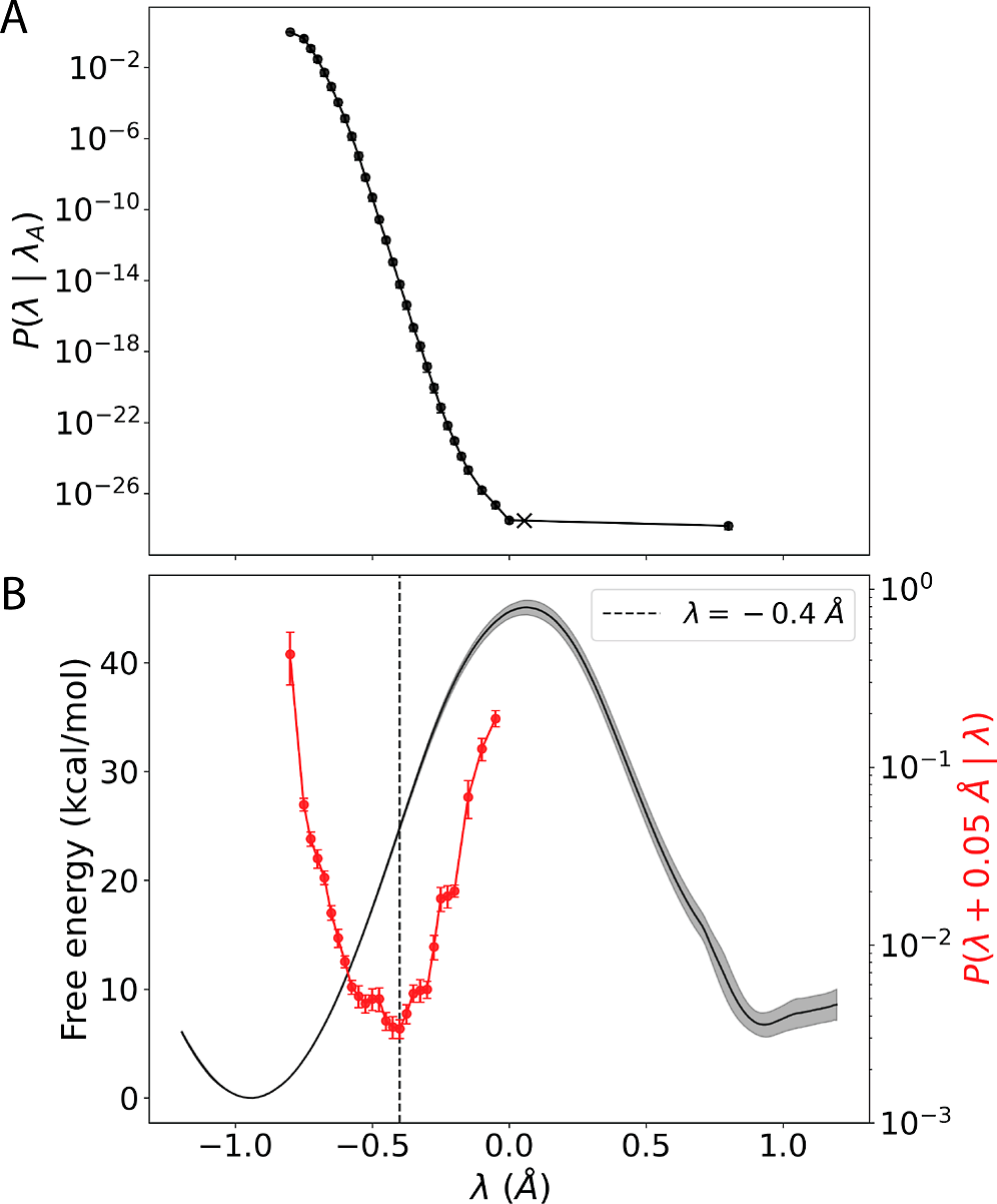
Characterization of WT reaction with kinetic and equilibrium methods. (A) Cumulative probability of the reaction complex reaching a certain level of reaction progress, given that the complex began in the reactant well (*P*(λ|λ_A_)). The region where λ < −0.8 Å = λ_A_ is considered the reactant well and is referred to as state A. The region in which λ > 0.8 Å = λ_B_ is considered the product well and is referred to as state B. Circle markers indicate the average and error bars designate ± SEM (*n* = 9 independent TIS rate calculations). (B) The detailed PMF curve (see “detailed mode” in Potential of Mean Force Calculations in the Methods section) along order parameter, λ (black line). The average profile is plotted and the shaded region indicates ± SEM (*n* = 3 independent PMF calculations). The probability (red line) of an attempted reaction making incrementally further progress along λ given that a certain level of progress was already made (*P*(λ + 0.05 Å|λ)). Circle markers indicate the average and error bars designate ± SEM (*n* = 9 independent TIS rate calculations). The dashed line indicates the location of the kinetically determined bottleneck near λ = −0.4 Å.

To inform the design of mutants with improved turnover, we first characterized the kinetics and reaction profile of the isomerization step catalyzed by WT KARI by using kinetic and equilibrium methods. Kinetic TIS-based rate calculations were used to compute the rate constant, *k*_cat_, which was computed to be (1.0 ± 0.4) × 10^–16^ s^–1^ (average ± SEM) across nine independent TIS calculations (Figure 3). Figure 3 shows representations of the probability of the reaction continuing (versus being turned back) as a function of the progress variable, λ. To determine how reaction progress related to the reaction energy barrier, the potential of mean force (PMF) along λ was calculated using umbrella sampling with the weighted histogram analysis method (WHAM, an equilibrium method; Figure 3B, black line). The free-energy peak was between λ = 0.065 and λ = 0.075 Å, suggesting that the TS occurred within this region, and the free energy of activation was 45.1 ± 0.7 kcal/mol (average ± SEM, *n* = 3 replicate calculations). Figure 3A indicates, on a log scale, a steady and continual loss of reaction progress between λ = −0.65 and −0.15 Å, during which the cumulative probability of the reaction continuing dropped by about 20 orders of magnitude. That is, the trajectories were turned back continuously throughout this range of λ values. Comparing to the free energy barrier profile in Figure 3B, this corresponds to most of the ramp leading to the top of the free energy barrier. Interestingly, the rapid loss of reaction progress did not continue to the top of the reaction barrier but ended somewhat earlier, as the probability of reverting to the reactant tracked well with the gradient of the free energy with respect to λ. Figure 3B (red line) shows the probability of the reaction continuing as a function of its progress. Figure 3 indicates that even though there was a relatively constant drop-off in reaction progress on the incline slope, the greatest drop-off occurred at a bottleneck near λ = −0.4 Å. That is, when the enzyme–substrate conformation reached λ ≈ −0.4 Å, it was least likely to make another 0.05 Å of progress along λ toward the product well. Put differently, the point along the route from reactant to product where an attempted reaction was most likely to fail and begin a return to the reactant well occurred near λ = −0.4 Å, which is about halfway up the incline of the energy barrier.

### ML Models Identify Structural Determinants of Productive Reactions

**Figure 4:**
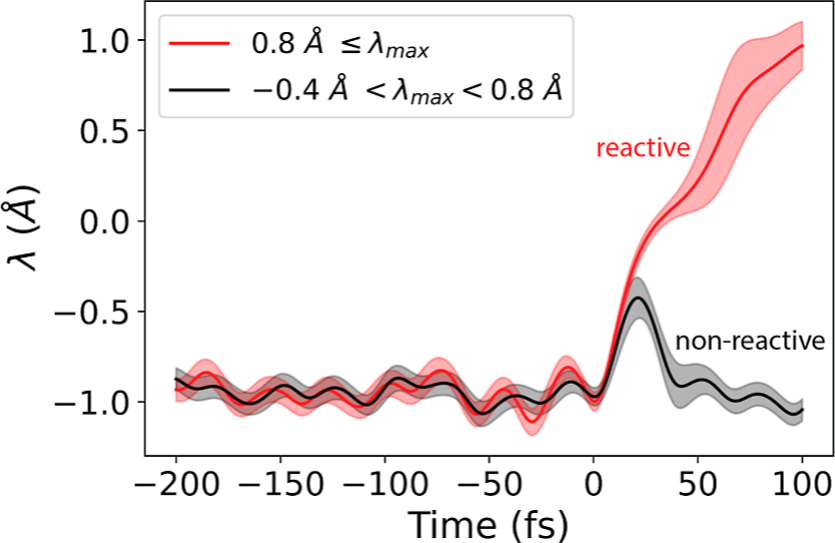
Simulation of reactive (R) and nonreactive (NR_–0.4_) trajectories. TIS was used to sample attempted turnover events in which the enzyme makes a prescribed level of progress (λ_max_) along the reaction. Two types of attempts, or pathway types, are shown: one being reactive (red) and the other nonreactive (black), which was required to reach at least λ > −0.4 Å before returning to the enzyme–substrate reactant well. The order parameter, λ, is plotted as a function of time. Lines indicate the average and shading indicates ±1 SD across all paths of that type.

Machine learning (ML) was used to identify structural prereaction characteristics that distinguished reactive (R) from nonreactive (NR) trajectories. The objective was to train models that distinguished between successful and failed reactions based on structural features describing enzyme–substrate conformations at early time points well before the attempted turnover event. In constructing this analysis, we chose to use NR pathways that were representative of failures occurring at the identified bottleneck near λ = −0.4 Å. This represented the point where a given reaction attempt was most likely to fail and bounce off of the energy barrier, beginning its return to the reactant well. We hypothesized that models trained to distinguish between such nearly reactive pathways and productive reactive pathways would be helpful in guiding enzyme redesign because they would highlight structural features that best support the reaction complex’s progress through the bottleneck. As such, 20 pathway ensembles were constructed for each pathway type: reactive paths (R) that reached the product well and nonreactive paths (NR_–0.4_) that reached at least λ = −0.4 Å before returning to the reactant well (Figure 4). For each pathway type (R and NR_–0.4_), the 20 pathway ensembles were evenly distributed across 10 unique starting seed pathways, and 10,000 shooting move attempts were used to construct each ensemble. The average shooting move acceptance rate was 24.6 and 28.4% for NR_–0.4_ and R pathways, respectively, generating 49,251 unique NR_–0.4_ pathways and 56,811 unique R pathways.

**Figure 5:**
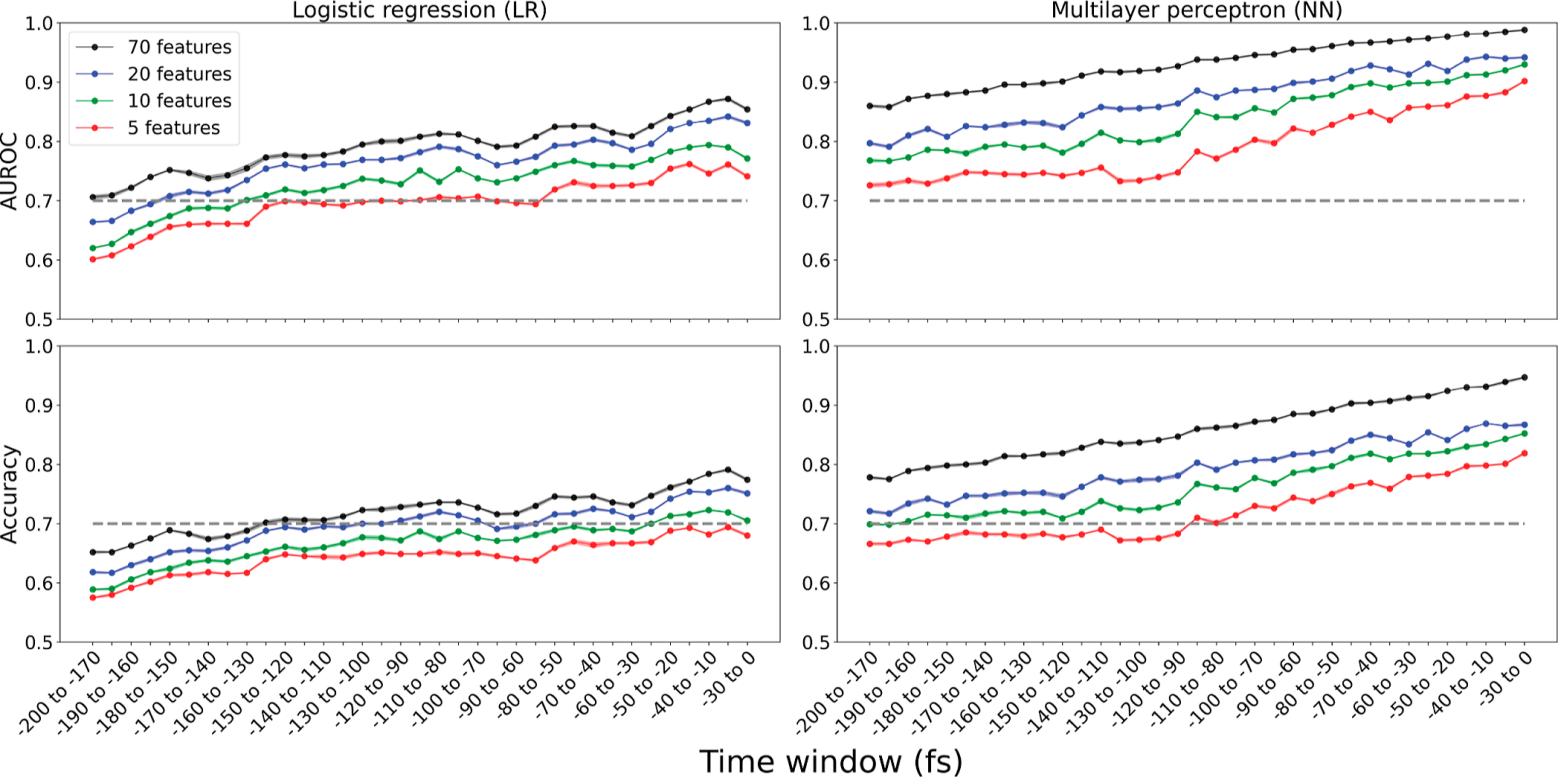
ML models predict whether an enzyme–substrate complex will successfully react and reach the product well. AUROC (top row) and accuracy (bottom row) of logistic regression (LR, left column) and multilayer perceptron (NN, right column) models trained and tested on conformations sampled within different 30-fs-long time windows. The models make predictions using a set of up to 70 structural features (interatomic distances, angles, and torsions) describing each conformation. Results are shown for unseen data using fivefold cross validation. Circle markers indicate the average, and shading indicates ± SEM (*n* = 5 folds).

Both logistic regression (LR) and multilayer perceptron (NN, “neural net”) models were trained to predict whether prereaction complexes were reactive (would later successfully reach the product well) or nonreactive (would later fail to reach product and would instead return to the reactant well). These predictions were made using up to 70 structural features (interatomic distances, angles, and torsions) describing the substrate-bound active site (Table S1). The training and testing data were comprised of conformations randomly sampled from time points within 30-fs-long time windows. In choosing this strategy, we sought characteristics describing regions of the reactant well that were more favorable for reactivity in general, and whose importance did not necessarily depend on the precise timing of passing through these regions on approach to the barrier. This contrasts with our previous work, in which all data used for training a particular model came from points corresponding to the same time before climbing the reaction barrier.^9^ To monitor predictive performance over time, a 30-fs-long time window was shifted in increments of 5 fs from a minimum of −200 fs to a maximum of 0 fs (the time corresponding to the last compression of the putative breaking bond), and new models were trained for each window placement. This choice of 30 fs was made based on the time scale of the oscillations of the bond vibrations in the substrate and its local neighborhood. For each time window, we applied feature selection strategies to limit the number of input features in order to identify the most predictive feature subsets and to evaluate the feasibility of training accurate models with fewer than 70 features (Figure 5).

When using all 70 features, LR models achieved an AUROC of 0.706 (65.2% accuracy) as far back as −200 to −170 fs. The predictive power increased up to 0.854 AUROC (77.4% accuracy) as the time window’s placement approached *t* = 0, and this performance decreased as fewer features were used (Figure 5, left column).

The NN models outperformed the LR models for all time points, achieving an AUROC as high as 0.988 (94.7% accuracy) when using all 70 features within the latest time window (−30 to 0 fs). Even as far back as −200 to −170 fs, NN models achieved an AUROC of 0.860 (77.8% accuracy) when using all 70 features and still surpassed AUROC > 0.70 when using only 5 features (Figure 5, right column).

### Protein Redesign Algorithm Generates Mutants with Increased Populations of Reactive-Like Conformations

**Figure 6:**
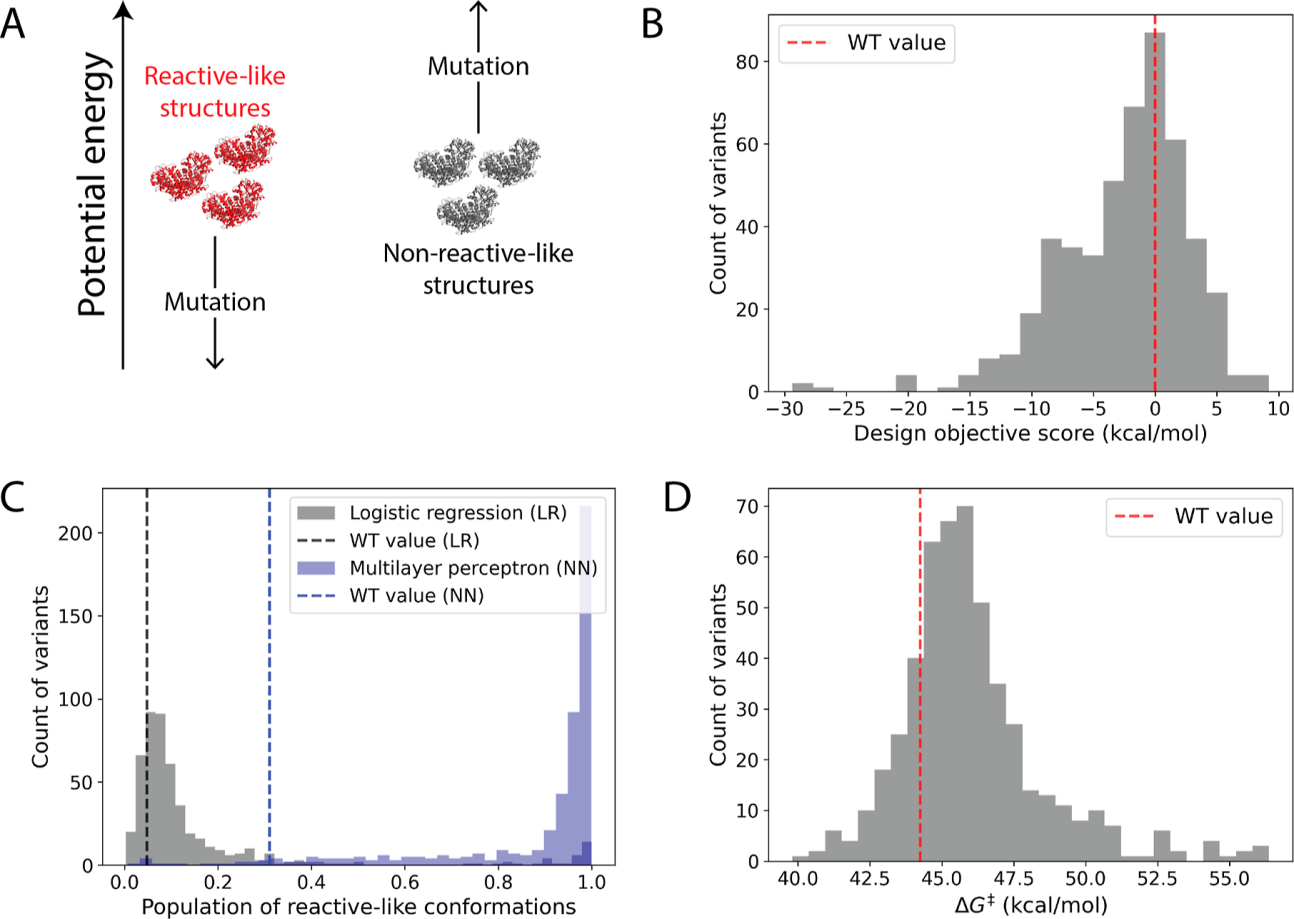
Selecting mutations to stabilize reactive-like structures relative to nonreactive-like ones. (A) Schematic depicting the COMETS design goal of energetically stabilizing the selected reactive-like structures (red) relative to the nonreactive-like (NR_–0.4_) ones (gray), which were chosen among truly reactive and nonreactive structures using trained LR and NN models. (B) Distribution of design objective scores, which indicate the energetic stabilization of the reactive-like structures relative to the nonreactive-like ones taken with respect to the corresponding WT structures; by construction, the WT values are 0 kcal/mol. If a mutant was selected in more than one design round, only its lowest (i.e., most favorable) score is counted in the histogram. For visual clarity, 11 outlier mutants with design objective scores below −30 kcal/mol were not included in the plot. (C) Distribution of unique enzyme variants’ average fraction of time inside the reactant well (at equilibrium) spent sampling conformations that were classified as reactive-like by the LR model (gray) or NN model (blue); averages were taken across *n* ≥ 3 independent simulations. (D) Distribution of unique enzyme variants’ Gibbs free energy of activation values (Δ*G*^‡^) taken from approximated PMF curves (see “screening mode” in Potential of Mean Force Calculations in the Methods section). WT values are indicated by dashed lines throughout.

After collecting and comparing reactive and nonreactive transition pathways in WT KARI, we used these as input for a newly developed design pipeline based on the hypothesis that mutants designed to stabilize reactive-like conformations over nonreactive-like ones relative to WT could lead to enhanced catalytic activity (*k*_cat_, specifically). The multistate DEE/A*-based protein design algorithm COMETS,^57^ implemented in OSPREY 3.0,^58^ was used to select mutations according to this criterion. To construct the design objective, subsets of representative reactive and nonreactive complex structures were gathered from the −160 to −130 fs window of WT transition pathways. For each subset, three structures were chosen from a pool of 3,287,922 using a procedure that was guided by the trained LR and NN models to specifically select enzyme–substrate complexes that were consistent with the reactive-like or nonreactive-like structural characteristics identified by the models (see the Methods section). With the selected subsets, in a given design round, COMETS was used to select mutations that energetically stabilized the reactive structures relative to the nonreactive ones by considering the energetically most favorable conformations of each sequence in each of the six structures (Figure 6A). A mutation was collected if the total difference between the sums of its reactive-like and nonreactive-like structures’ energies was within 20 kcal/mol of the optimal sequence (this means that COMETS could still select mutations that did worse than WT), and if the sequence’s reactive-like structures were no more than 5 kcal/mol less stable than the WT sequence’s. This constrained design objective was intended to select mutations that favored reactive-like over nonreactive-like structures relative to WT (allowing some leeway), without relying on significant destabilization of reactive-like structures to do so. Ultimately, we intended to select only mutants with negative (i.e., favorable) design objective scores for further study, but we cast a wider net at this stage.

In total, we report results compiled from four different design rounds, which tested different strategies for choosing reactive and nonreactive structures in the design objective function, and the final design round searched for double mutants (see the Methods section). In this broad initial selection, 715 variants were designed, of which 502 were unique (some variants were uncovered in more than one design round). Among the unique variants, 340 (67.7%) showed increased stabilization of reactive-like structures over nonreactive-like structures (Figure 6B), as evaluated by their potential energies. The remaining 32.3% had design objective scores within 20 kcal/mol of the best sequence. Each variant’s proclivity for sampling reactive-like conformations was further evaluated by tracking the frequency with which they adopted such conformations over the course of equilibrium dynamics simulations inside the reactant well (Figure 6C). The majority of the 340 mutants with favorable design objective scores more frequently adopted reactive-like conformations than WT: 270 (79.4%) showed an increase in the population of reactive-like conformations relative to nonreactive-like as evaluated by the LR model and 331 (97.4%) as evaluated by the NN model.

We explored the effectiveness of screening steps to remove mutants less likely to exhibit increased reactivity (larger *k*_cat_) and focused on more likely candidates in downstream, more expensive characterization. Given that our COMETS designs were informed only by conformations from before the complex climbed the reaction barrier, the design procedure received no direct information regarding structural and energetic changes along the actual event of the reaction. This means that while a designed mutant might have the intended effect of increased sampling of reactive-like conformations, it could also have unintended additional effects that disrupt the progress of pathways leading up and over the barrier so as to interfere with reactivity. To cull at least some variants with such unintended, detrimental effects, we estimated variants’ energy barriers by calculating an approximate PMF curve (see “screening mode” in Potential of Mean Force Calculations in the Methods section) along the order parameter, λ, and computing the Gibbs free energy of activation (Δ*G*^‡^). This analysis suggested that 99 redesigned variants (19.7%) had lower, more favorable energy barriers than did the WT (Figure 6D). Interestingly, the fraction of mutants with lower barriers than WT was increased among subsets of top-scoring candidates: 23.8% for the candidates in the top 20, and 29.4% among those in the top 10%. This means that the mutants with the most favorable design objective scores were generally more likely to have decreased energy barriers, even though the design scores were not informed by the reaction energy surface or TS-like structures. For screening candidate mutants for further testing, we considered using Δ*G*^‡^ values in addition to the design objective scores and the relative sizes of reactive-like conformation populations during equilibrium dynamics (as determined by both the LR and NN models). We did not initially define specific criteria based on these analyses, but rather we explored mutants with different values across these categories in order to evaluate which metrics would be most helpful in finding promising candidates.

### Redesigned Variants Showed Increased Activity

**Figure 7:**
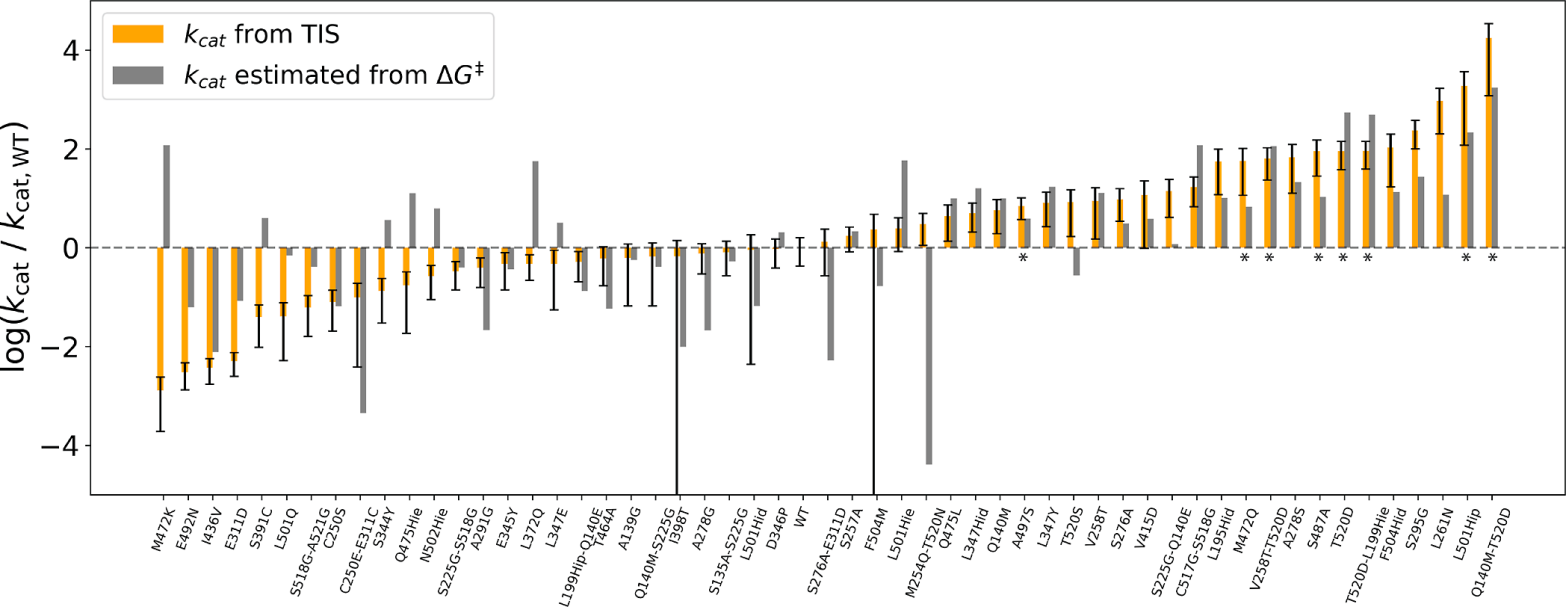
TIS-computed rate constants, *k*_cat_, for selected set of redesigned KARI variants composed of WT and 54 mutants. Orange bars indicate the ratio of mutant to WT *k*_cat_ and error bars span ± SEM (propagated error from the WT and mutant *k*_cat_ values, which were each averaged from *n* = 9 independent TIS rate calculations). Mutants with significant increases in TIS-calculated *k*_cat_ that were detected using the Benjamini-Hochberg^59^ procedure to control the false discovery rate (FDR) at α = 0.05 are marked with an asterisk. Approximated PMF curves (see “screening mode” in Potential of Mean Force Calculations in the Methods section) were calculated for each variant and used to estimate Δ*G*^‡^. Eyring equation-based *k*_cat_/*k*_cat,WT_ estimates from the Δ*G*^‡^ values are indicated by gray bars. Axis is shown in base-10 log scale. Hid and Hie indicate neutral histidine residues with the proton in the δ and ϵ positions, respectively. Hip indicates a doubly protonated, charged histidine residue.

In total, 54 redesigned mutants were tested for increased activity by computing their *k*_cat_ values with our full TIS rate calculation. Rather than selecting these 54 variants using strict criteria based on increased populations of reactive-like conformations and/or reduced reaction barrier heights, we selected a varied set representing different barrier heights and tendencies for sampling reactive-like conformations. This choice was made to explore the space of designed mutations and to determine which factors were most indicative of successful variants. Twenty-eight (52%) of the tested mutants had larger computed *k*_cat_ values than WT (Figure 7). Among these, 26 (93%) more frequently populated reactive-like conformations than WT and, based on approximate PMF calculations used for screening, all but four had energies of activation (Δ*G*_MUT_^‡^) that were either comparable to or less than WT’s (Δ*G*_WT_^‡^). Among the 26 mutants with calculated *k*_cat_ values smaller than that of WT, only 19 (73%) showed increased sampling of reactive-like conformations compared to WT, and 13 of these 19 were computed to have a higher energy of activation than WT, suggesting that a majority of the slower-than-WT variants may have lost the benefit of their increased reactive-like conformation sampling through barrier height increases. Of the seven slower-than-WT variants with decreased reactive-like conformation sampling compared to WT, five had a higher energy barrier than WT. Among the set of 28 variants with both a comparable or reduced barrier height and an increased population of reactive-like conformations compared to WT, 22 (79%) had larger computed *k*_cat_ than WT. In contrast, within the set of five variants with an increased energy barrier and a reduced population of reactive-like conformations, none had larger calculated *k*_cat_ than WT (Figure 7). Taken together, these results demonstrate that reductions in barrier height and increases in reactive-like conformational sampling tended to occur together among the selected mutants, and they tended to lead to increased computed *k*_cat_. Mutants with increased sampling of reactive-like conformations tended to lead to increased *k*_cat_ unless the barrier height increased as well.

The Benjamini–Hochberg procedure was used to select high-confidence mutants with increased activity while controlling the false discovery rate (FDR) at α = 0.05, resulting in the selection of eight mutants with statistically significant increases in the computed rate constant (Figure 7). There were 20 other mutants identified with increased computed *k*_cat_ relative to WT, but due to statistical uncertainty, one cannot be as confident that they are truly computed faster than WT.

### Multiple Mechanisms Explain Fast Mutants’ Rate Enhancements

Among the 54 mutants that were tested for increased activity by TIS rate (*k*_cat_) calculations, we pursued a further structural analysis of the eight high-confidence fast mutants to understand the basis for their rate enhancement. We first compared the relative sizes of populations of reactive-like structures at equilibrium (GS) and while on the approach to attempted reactions (dynamic), as evaluated by the LR model and separately the NN model (Table 1).

**Table 1:** Populations of Reactive-Like Conformations in Different Enzyme Variants^a^

variant	GS [LR]	NR_–0.4_ [LR]	R [LR]	GS [NN]	NR_–0.4_ [NN]	R [NN]
WT	1.0 ± 0.1 (0.048 ± 0.005)	1.0 ± 0.2 (0.35 ± 0.05)	1.00 ± 0.08 (0.64 ± 0.04)	1.00 ± 0.07 (0.31 ± 0.01)	1.00 ± 0.05 (0.147 ± 0.005)	1.00 ± 0.01 (0.842 ± 0.009)
Q140M-T520D	3.2 ± 0.5 (0.15 ± 0.02)	0.9 ± 0.2 (0.33 ± 0.05)	0.7 ± 0.1 (0.46 ± 0.07)	1.60 ± 0.09 (0.50 ± 0.01)	4.0 ± 0.5 (0.59 ± 0.07)	0.93 ± 0.06 (0.79 ± 0.05)
L501H	2.6 ± 0.3 (0.126 ± 0.008)	0.8 ± 0.2 (0.27 ± 0.05)	0.75 ± 0.06 (0.48 ± 0.03)	3.2 ± 0.2 (0.990 ± 0.004)	6.8 ± 0.2 (0.9992 ± 0.0005)	1.19 ± 0.01 (0.9993 ± 0.0002)
T520D-L199H	8 ± 1 (0.37 ± 0.04)	1.6 ± 0.3 (0.55 ± 0.08)	1.0 ± 0.1 (0.63 ± 0.05)	3.1 ± 0.2 (0.974 ± 0.006)	6.8 ± 0.2 (0.9991 ± 0.0007)	1.19 ± 0.01 (0.9997 ± 0.0001)
T520D	6.3 ± 0.6 (0.302 ± 0.007)	1.6 ± 0.3 (0.56 ± 0.06)	1.03 ± 0.08 (0.67 ± 0.03)	1.60 ± 0.08 (0.50 ± 0.01)	3.4 ± 0.3 (0.50 ± 0.04)	0.89 ± 0.04 (0.75 ± 0.03)
S487A	2.1 ± 0.2 (0.102 ± 0.005)	1.6 ± 0.3 (0.55 ± 0.06)	1.0 ± 0.1 (0.64 ± 0.05)	1.8 ± 0.6 (0.6 ± 0.2)	4.9 ± 0.8 (0.7 ± 0.1)	0.98 ± 0.09 (0.83 ± 0.07)
V258T-T520D	9 ± 1 (0.42 ± 0.02)	1.5 ± 0.2 (0.52 ± 0.03)	1.10 ± 0.09 (0.71 ± 0.04)	3.2 ± 0.2 (0.9960 ± 0.0004)	6.8 ± 0.2 (0.9994 ± 0.0004)	1.19 ± 0.01 (0.9998 ± 0.0001)
M472Q	1.7 ± 0.2 (0.083 ± 0.003)	0.7 ± 0.1 (0.24 ± 0.02)	0.83 ± 0.06 (0.53 ± 0.03)	3.1 ± 0.2 (0.98 ± 0.02)	6.8 ± 0.2 (0.9986 ± 0.0005)	1.19 ± 0.01 (0.9999 ± 0.0001)
A497S	3.5 ± 0.7 (0.17 ± 0.03)	1.5 ± 0.2 (0.52 ± 0.04)	1.08 ± 0.09 (0.70 ± 0.04)	1.3 ± 0.1 (0.40 ± 0.02)	3.2 ± 0.5 (0.46 ± 0.06)	0.87 ± 0.05 (0.73 ± 0.04)

a Values indicate the fraction of time steps populated by reactive-like conformations as identified by the LR or NN model, normalized by WT, with entries in parentheses indicating the unnormalized average ± SEM. For reactant well (GS) simulations, *n* = 3 independent calculations. For nonreactive (NR_–0.4_) and reactive (R) pathway simulations, data were used from the −160 to −130 fs window and *n* = 5 for mutants and *n* = 10 for WT.

Equilibrium simulations of GS dynamics indicated that all eight mutants more frequently populated reactive-like conformations than WT, as evaluated by both the LR and NN models, with increases ranging from 1.3- to 9-fold (Table 1). This shift in the equilibrium conformational ensemble was consistent with the design intent of selecting mutations that stabilized reactive-like conformations.

Comparing the equilibrium results to the dynamic results provides interesting insights. First, for WT, the fraction of time spent in the more productive part of the reactant well (i.e., sampling reactive-like conformations) was much larger for dynamic reactive trajectories than for equilibrium trajectories (64% vs 5% using the LR model and 84% vs 31% using the NN model). The models disagree for the comparison between dynamic nonreactive trajectories and equilibrium ones, with the LR model measuring an increase in the fraction of time in the more productive part of the reactant well in dynamic rather than equilibrium trajectories (35% vs 5%) and the NN model identifying a decrease (15% vs 31%). Taken together, these results show that equilibrium populations can be different from dynamic ones. In this context, our design strategy of selecting mutants based on their ability to stabilize specific structures populated along reactive trajectories relative to similar structures from nonreactive trajectories seems appropriate.

Moving now to the mutants, the dynamic behaviors show that some, but not all, of the mutants exhibited increased populations of the more productive portion of the reactant well in either the nonreactive or reactive trajectories, or both (5 out of 8 ranging from 1.5- to 1.6-fold as judged by the LR model and 8 out of 8 ranging from 3.2- to 6.8-fold by the NN model; Table 1). The results suggest that designs may have not only increased the desired population but also opened other pathways for both reactivity and nonreactivity.

Interestingly, for all the enzyme variants, both the NN and LR models reported larger reactive-like populations before reactive simulations than before nonreactive ones (in the −160 to −130 fs window, Table 1). This suggests that differences between reactive and nonreactive conformations, as learned from WT data, generalized and transferred to the mutants.

Collectively, these results indicate that the eight high-confidence mutants more frequently populated reactive-like structures than WT at equilibrium and largely before nonreactive turnover attempts, which was the initial design goal expected to increase catalytic activity. Interestingly, there were several mutants with smaller populations of reactive-like structures than WT in reactive pathways, and some of these mutants still had larger reactive-like populations than WT in nonreactive pathways. The observation that some faster-than-WT mutants populated reactive-like structures less frequently than WT in reactive pathways suggests that these mutants might have accessed new reaction channels that were not observed for WT. This indicates that the mutations, in addition to tuning the balance of catalytic features observed for WT, could also open new modes for catalytic failure or success. Taken together and combined with potential changes to the reaction energy landscape, these have a net effect on activity. Nonetheless, there were enough signals overall based on WT behavior to identify mutants with significantly increased *k*_cat_ compared to WT.

**Figure 8:**
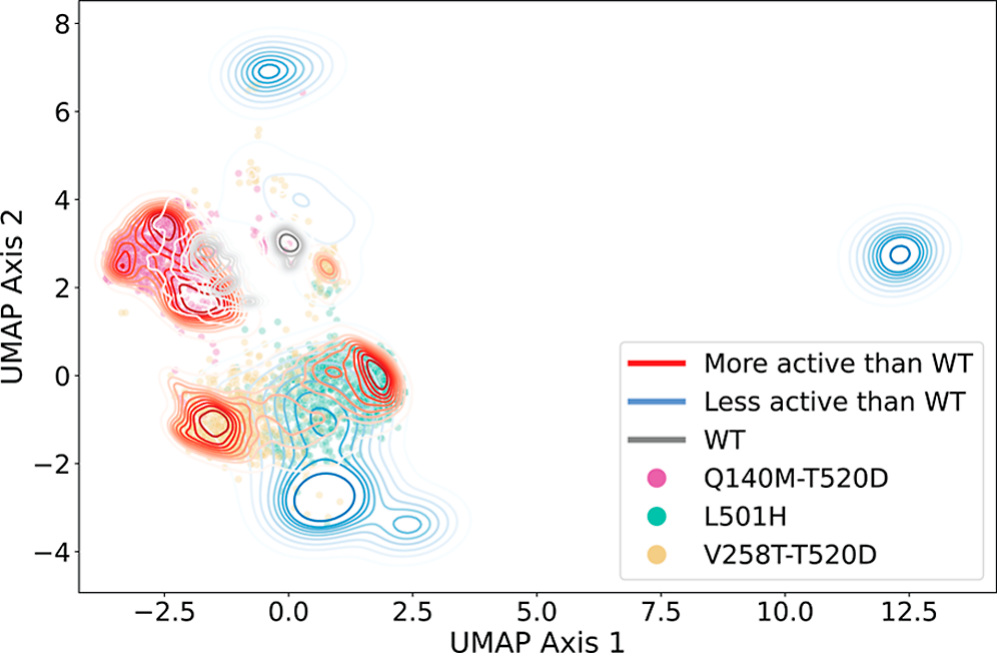
UMAP embedding of WT’s and designed variants’ prereaction enzyme–substrate conformations. For each variant, a representative subset of 1000 unique conformations was randomly sampled from 160 to 130 fs before *t* = 0 in transition pathways. The 70 interatomic features (distances, angles, and torsions) describing the structure of the active site were computed for each conformation, and these were embedded into two dimensions with UMAP. The distribution over the eight fast variants with significant increases in *k*_cat_, relative to WT, is shown in red. The distribution over a representative subset of eight variants with decreased *k*_cat_ compared to WT, the slowest eight variants overall, is shown in blue. The WT distribution is shown in gray. Embedded points for Q140M-T520D (purple), L501H (green), and V258T-T520D (yellow) are also indicated by circles.

To visualize how the eight high-confidence fast mutants were related with regard to their structural dynamics prior to attempted reactions, we computed a UMAP embedding of conformations that were sampled −160 to −130 fs before successful and failed turnover attempts (Figure 8). For this, we pooled conformations from the eight fast variants, WT, and a representative set of eight slower-than-WT variants. The embedding of these conformations largely separated the faster variants from the slower variants and uncovered three distinct groups among the faster mutants. The mutants that were embedded into the same “fast” group typically altered the same structural features (from the original set of 70 interatomic distances, angles, and torsions) in a similar fashion. Specific mutant vs WT comparisons are shown for Q140M-T520D, L501H, and V258T-T520D, which were chosen as representative examples of each of their respective clusters in the UMAP embedding. Interestingly, while Q140M-T520D and V258T-T520D have in common a T520D substitution, they were characterized differently in the embedding.

**Figure 9:**
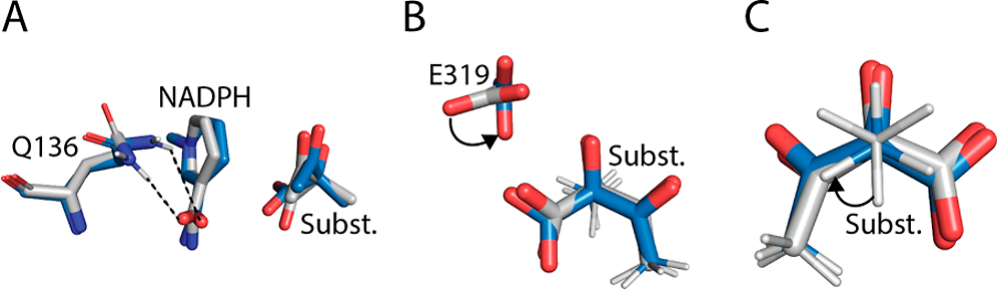
Reactive-like structural changes observed across several designed mutants. These changes correspond to the columns in Table 2. (A) The loss of the hydrogen bond, indicated with a dashed line, from Q136 to NADPH’s nicotinamide carbonyl was associated with reactive-like structures. (B) The rotation of E319, indicated by the curved arrow, was associated with reactive-like structures. (C) The eclipsing of migrating methyl, C_5_, against neighboring groups (here shown for groups bound to C_4_) was associated with reactive-like structures. The curved arrow indicates a transition from the staggered to eclipsed conformation. Representative structures were sampled from 160 to 130 fs before *t* = 0. Blue, reactive-like structure. Gray, nonreactive-like structure.

The three mutants representing the three UMAP clusters (composed of mutants with increased specific activity relative to WT) are analyzed and compared in the following paragraphs. The general strategy involved studying how their conformational dynamics affected any structural features originally used by the ML models to distinguish between WT’s R and NR_–0.4_ conformations. This analysis identified three unique and independent reactive-like structural characteristics that could explain the enhanced activity of the fast mutants: (i) the loss or weakening of a hydrogen bond from Q136 to NADPH, (ii) the rotation of E319’s carboxylate group, and (iii) the eclipsing of the migrating methyl against neighboring groups in the substrate (Figure 9). The frequencies of these three features were quantified in transition pathways from −160 to −130 fs for each enzyme variant (Table 2).

**Table 2:** Frequency of Reactive-Like Structural Characteristics in Different Enzyme Variants^a^

variant	no Q136-NADPH H-bond	rotated E319	eclipsed C5
WT	1.000 (0.521)	1.000 (0.142)	1.000 (0.239)
Q140M-T520D	0.656 (0.341)	5.654 (0.801)	1.215 (0.290)
L501H	1.593 (0.829)	0.526 (0.075)	1.520 (0.363)
T520D-L199H	1.811 (0.943)	6.288 (0.891)	0.979 (0.234)
T520D	0.850 (0.442)	3.860 (0.547)	1.329 (0.318)
S487A	1.265 (0.659)	0.704 (0.100)	1.279 (0.306)
V258T-T520D	1.810 (0.942)	6.125 (0.868)	0.814 (0.195)
M472Q	1.221 (0.636)	0.381 (0.054)	1.400 (0.334)
A497S	1.394 (0.726)	1.472 (0.209)	1.545 (0.369)

a Values indicate the fraction of conformations consistent with the reactive-like feature in the −160 to −130 fs time window across both reactive (R) and nonreactive (NR_–0.4_) pathways. Measurements are normalized by WT with raw values reported in parentheses (see Methods).

**Figure 10:**
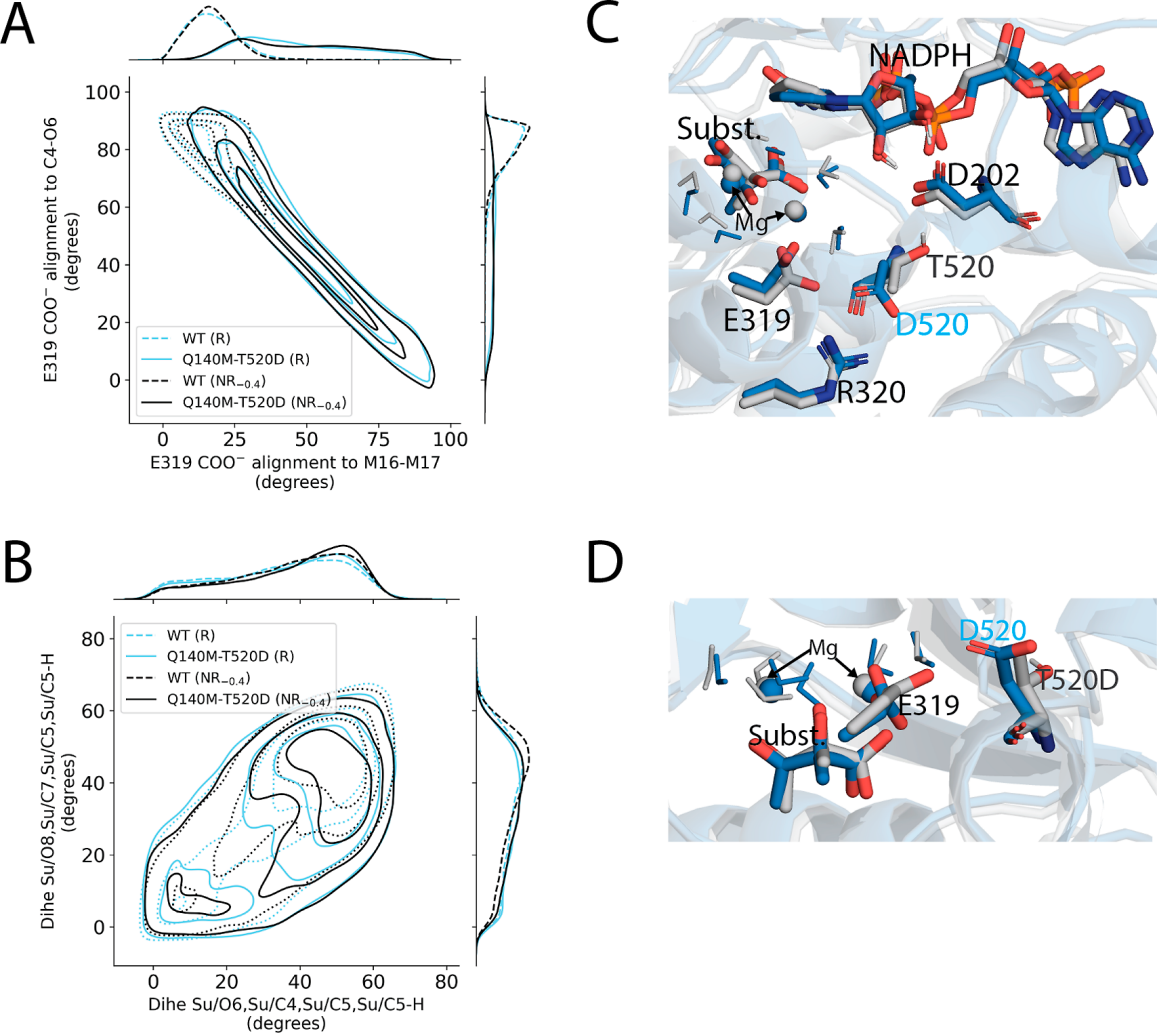
Conformational changes observed near the active site of Q140M-T520D. (A) Contour plot of the alignment between the E319 carboxylate and the axis joining the Mg^2+^ ions (*x*-axis) vs the axis joining the substrate atoms C_4_ and O_6_ (*y*-axis). Alignments are reported as the angle between the two respective axes such that values closer to zero indicate closer alignment. (B) Contour plot of the dihedral angles describing the overlap of the migrating methyl’s three hydrogens to substrate atoms O_6_ (*x*-axis) and O_8_ (*y*-axis). Lower values indicate closer, more eclipsed-like, alignment. (C,D) Representative enzyme–substrate structures for WT (gray) and Q140M-T520D (blue). The mutant D520 formed a salt bridge with R320, maintaining a conformation that crowded E319, whereas the WT T520 participated in a hydrogen bond network involving D202, NADPH, and Mg^2+^ ion-coordinating waters. Each contour plot distribution was constructed from a representative set of 10,000 unique enzyme–substrate conformations sampled within the −160 to −130 fs window in reactive (R) or nonreactive (NR_–0.4_) pathways.

The fastest variant was Q140M-T520D with a computed *k*_cat_ of (2 ± 1) × 10^–12^ s^–1^ (average ± SEM), which was 20,000 times faster than WT. There were several structural features implicated in Q140M-T520D’s enhanced activity (Figure 10), as evidenced by their shifts away from WT’s nonreactive-like values and toward reactive-like values. The most distinctive changes included (i) a 5.7-fold increase in the population of a rotated E319 conformation (Figure 10A) and (ii) a 21.5% increase in the frequency of the migrating methyl, C_5_, eclipsing against its neighboring groups bound to either C_4_ or C_7_ in the substrate (Figure 10B). Both of these structural characteristics were associated with successful turnover in WT: relative to NR_–0.4_ pathways, the rotated E319 and eclipsed C_5_ conformations were increased by 39.8% and 12.4% in R pathways, respectively.

Q140M-T520D exhibited two different E319 conformations: one with the axis spanning the two E319 carboxylate oxygens being aligned with the axis from substrate atoms C_4_ to O_6_, and one with these two axes being roughly perpendicular (Figure 9B). We refer to the aligned conformation, which was associated with increased catalytic efficiency, as “Rotated E319.” In WT, the rotated E319 conformation was rare (i.e., the axis joining C_4_ to O_6_ was generally perpendicular to the axis joining E319’s carboxylate oxygens. The angle between these two axes was less than 75° in only 14.2% of WT conformations, whereas, for Q140M-T520D, this angle was less than 75° in 80.1% of conformations (Table 2). This rotation of catalytic E319 propagated effects further across the active site, as E319 interacted directly with both the substrate and the Mg^2+^ ion cofactors and coordinating waters. Indeed, many of the features describing the relative positions of substrate, Mg^2+^ ions, and Mg^2+^-coordinating waters were favorably shifted toward reactivity (i.e., toward values that were more common in WT R pathways than NR_–0.4_ pathways) in Q140M-T520D, though the magnitudes of these shifts were not as pronounced and distinctive as the rotation of E319’s carboxylate. All other fast variants that contained a T520D mutation showed similar E319 behavior as well as concomitant perturbations to the features describing the relative positions of the substrate, Mg^2+^ ions, and Mg^2+^-coordinating waters, which suggests the new E319 conformation was caused by the T520D mutation. Residue 520 neighbored E319, and these two residues participated together in a network of hydrogen bonds and electrostatic interactions (Figure 10C). In WT, the T520 residue donated a hydrogen bond to D202, maintaining T520 in a conformation that allowed more space for E319 to align its carboxylate oxygens’ axis with the axis between the Mg^2+^ ions, which was roughly perpendicular to the axis between C_4_ and O_6_. In Q140M-T520D, however, the D520 side chain formed a salt bridge with R320, which pulled D520's carboxylate within the vicinity of E319 and the resulting electrostatic repulsion contributed to a 5.7-fold increased adoption of the rotated E319 conformation (Figure 10C,D). Previous studies from our group have implicated E319’s mechanistic role in KARI’s methyl transfer reaction.^48^

Q140M-T520D’s migrating methyl, C_5_, adopted eclipsed conformations against the groups bonded to either C_4_ or C_7_ 21.5% more often than WT’s did (Table 2). Eclipsed conformations were characteristic of successful catalysis in WT, and their increased adoption by Q140M-T520D could thus contribute to the calculated increase in *k*_cat_. The eclipsing of C_5_ against neighboring groups was previously linked to KARI catalysis in a separate study by our group.^50^

**Figure 11:**
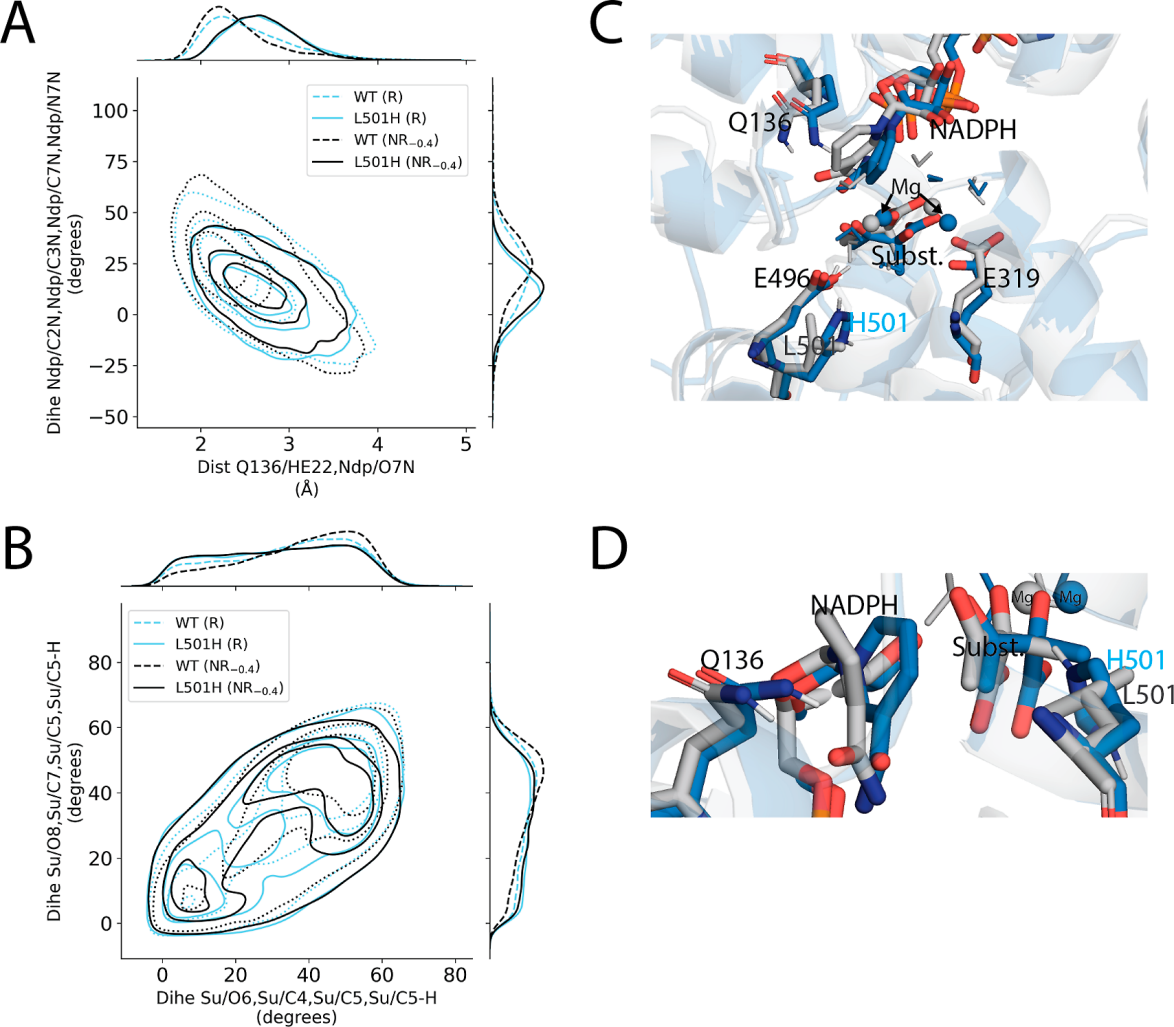
Conformational changes observed near the active site of L501H. (A) Contour plot reporting on the strength of the putative hydrogen bond from Q136 to NADPH’s nicotinamide (hydrogen bond distance on the *x*-axis) and the planarity of the nicotinamide ring (dihedral angle on the *y*-axis where values closer to zero indicate a more planar arrangement). (B) Contour plot of the dihedral angles describing the overlap of the migrating methyl’s three hydrogens to substrate atoms O_6_ (*x*-axis) and O_8_ (*y*-axis). Lower values indicate closer, more eclipsed-like, alignment. (C,D) Representative enzyme–substrate structures for WT (gray) and L501H (blue). The mutant H501 (doubly protonated) introduced a new positive charge within the active site, and the hydrogen bond between Q136 and NADPH was weaker in L501H than WT. Each contour plot distribution was constructed from a representative set of 10,000 unique enzyme–substrate conformations sampled within the −160 to −130 fs window in reactive (R) or nonreactive (NR_–0.4_) pathways.

The second fastest variant was L501H (specifically a doubly protonated, cationic H501 in the model), with a computed *k*_cat_ of (2 ± 1) × 10^–13^ s^–1^ (average ± SEM), 2000 times faster than WT. Again, there were two key structural features implicated in L501H’s enhanced activity (Figure 11), as evidenced by their shifts toward WT’s reactive-like values: (i) NADPH’s interactions with Q136 and substrate (Figure 11A) and (ii) an increased eclipsing of the migrating methyl, C_5_, against its neighboring groups in the substrate (Figure 11B). The mutation introduced a new positive charge in the active site at residue 501 which directly interacted with catalytic residue E496 and substrate, with electrostatic interactions with NADPH and E319 also possible (Figure 11C). The rotated E319 conformation characteristic of T520D-containing mutants was not observed for L501H (Table 2), suggestive of a model in which there are multiple reactive channels within the space of reactive transition pathways such that there are many supporting interactions that can enhance reaction rate, of which some amount, but not all, are needed.

For WT, the presence of a hydrogen bond from Q136 to NADPH’s amide was observed in the crystal structure as well as its energetically minimized and equilibrated structures. However, the hydrogen bond was associated with failed turnover attempts. The 47.9% frequency of this hydrogen bond in WT’s transition pathway conformations was reduced to 17.1% in L501H, and its loss was correlated with a more planar arrangement of the nicotinamide ring and a shorter distance from the nicotinamide carbonyl to the substrate carboxylate (Table 2 and Figure 11A). While these secondary changes were typically associated with the loss of the hydrogen bond from Q136 to NADPH’s amide, we chose to focus mainly on the hydrogen bond as the indicator of this class of changes because the hydrogen bond was a noticeable structural difference that we independently observed across several enzyme mutants. The L501H mutation introduced a new positive charge about seven Å from the nicotinamide carbonyl and opposite to Q136. This positioning could draw the nicotinamide carbonyl oxygen away and contribute to its lost interaction with Q136. This structural shift was associated with reactivity because the population of conformations without the Q136 to NADPH hydrogen bond was increased by 34.4% in WT R trajectories relative to NR_–0.4_ ones (Figure 11D).

Compared to WT, L501H more frequently populated conformations with the migrating methyl C_5_, eclipsing the groups bonded to C_4_ and C_7_ (Figure 11B). Relative to WT, the population of eclipsed C_5_ conformations was increased by 52.0% (Table 2), and such eclipsed conformations were related to successful substrate conversion in WT. The eclipsing against C_4_’s groups is suggestive of a higher energy substrate immediately prior to an attempted reaction, and it may also have electronic structural reasons for facilitating product formation.

**Figure 12:**
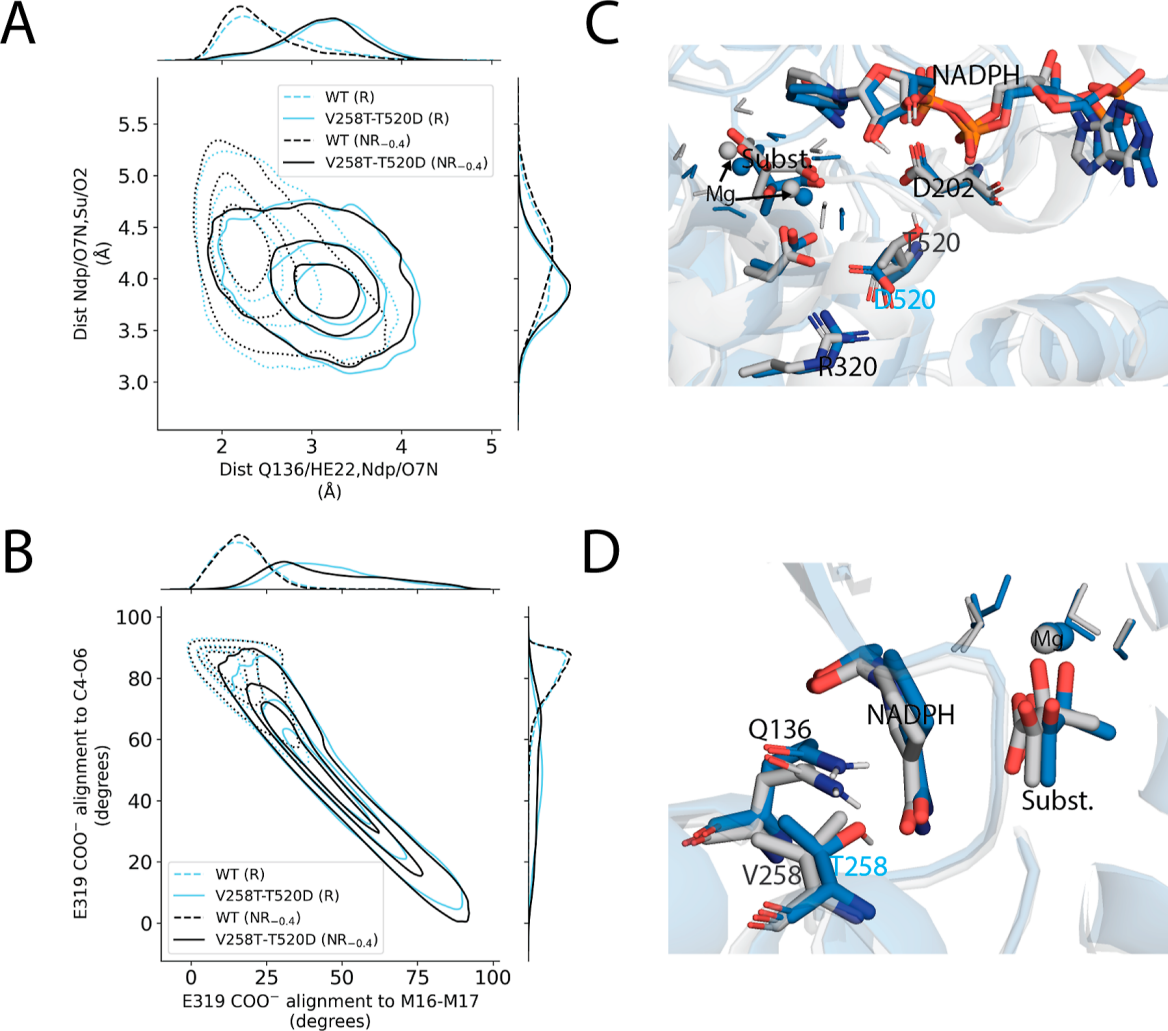
Conformational changes observed near the active site of V258T-T520D. (A) Contour plot reporting on the strength of the putative hydrogen bond from Q136 to NADPH’s nicotinamide (hydrogen bond distance on the *x*-axis) and the distance between the nicotinamide oxygen and substrate atom O_2_ (*y*-axis). (B) Contour plot of the alignment between the E319 carboxylate and the axis joining the Mg^2+^ ions (*x*-axis) versus the axis joining the substrate atoms C_4_ and O_6_ (*y*-axis). Alignments are reported as the angle between the two respective axes such that values closer to zero indicate closer alignment. (C,D) Representative enzyme–substrate structures for WT (gray) and V258T-T520D (blue). (C) The mutant D520 formed a salt bridge with R320, maintaining a conformation that crowded E319, whereas the WT T520 participated in a hydrogen bond network involving D202, NADPH, and Mg^2+^ ion-coordinating waters. (D) The V258T mutation introduced a hydroxyl group within the first interaction shell of Q136 and opposite from NADPH. T258 could accept a hydrogen bond from Q136 and thereby compete with NADPH’s nicotinamide for interaction with Q136. Each contour plot distribution was constructed from a representative set of 10,000 unique enzyme–substrate conformations sampled within the −160 to −130 fs window in reactive (R) or nonreactive (NR_–0.4_) pathways.

Embedded separately from Q140M-T520D and L501H (Figure 8), V258T-T520D was the 10th fastest variant overall (*k*_cat_ = (6 ± 3) × 10^–15^ s^–1^, average ± SEM) and 60 times faster than WT. Like the previous two mutants, its increased catalytic efficiency was primarily attributed to two structural characteristics (Figure 12): (i) a weaker interaction between Q136 and NADPH’s nicotinamide (Figure 12A) and (ii) an increase in the population of the rotated E319 conformation (Figure 12B). As previously described for Q140M-T520D, the T520D mutation was believed to be linked to the rotated E319 conformation (Figure 12C). Critically, however, the reduced interaction between Q136 and NADPH was only observed in two of the four T520D-containing mutants (of which Q140M-T520D was not one) and, in this case, was believed to be directly attributed to the V258T substitution.

The V258T mutation introduced a new hydroxyl group within the first interaction shell of Q136 and opposite from NADPH’s nicotinamide (Figure 12D). The mutant T258 residue could accept a hydrogen bond from Q136 and competed for its interaction against NADPH’s nicotinamide. This had the effect of reducing the WT’s 47.9% frequency of the hydrogen bond between Q136 and NADPH’s amide to 5.8% in V258T-T520D, which again correlated with a slightly more planar nicotinamide geometry and a shorter distance from NADPH’s amide carbonyl to the substrate carboxylate (Figure 12A). The population of conformations without the Q136 to NADPH hydrogen bond was increased by 34.4% in WT R pathways relative to NR_–0.4_ pathways, which implicated this structural change in V258T-T520D’s increased catalytic efficiency.

Similar to Q140M-T520D, V258T-T520D often exhibited the rotated E319 conformation, in which the axis joining the two E319 carboxylate oxygens was more parallel to the axis joining substrate atoms C_4_ to O_6_ (Figure 12B); this general characteristic of T520D-containing variants was observed 6.1-fold more frequently for V258T-T520D than for WT (Table 2). E319 interacted directly with the substrate, Mg^2+^ ions, and Mg^2+^-coordinating waters, and the structural features involving these groups were again favorably shifted toward values correlated with successful turnover, similar to observations for other T520D-containing mutants. The mutated residue 520, again, explains the altered E319 dynamics. In WT, T520 donated a hydrogen bond to D202. V258T-T520D’s D520 instead formed a salt bridge with R320, and this positioning of the negatively charged D520 side chain destabilized the WT-preferred E319 conformation by electrostatic repulsion (Figure 12C). These observations match those for Q140M-T520D.

Unlike Q140M-T520D and L501H, V258T-T520D was not expected to derive any of its catalytic advantage over WT (and may even have paid a cost) from the migrating methyl’s eclipsing of nearby groups in the substrate, with an observed 18.6% decrease in the frequency of such conformations relative to WT (Table 2).

The examination of R and NR_–0.4_ trajectories in Q140M-T520D, L501H, and V258T-T520D revealed three general structural changes associated with increased levels of catalytic activity: the loss of the hydrogen bond between Q136 and NADPH, the rotated E319 conformation, and the increased level of eclipsing of the migrating methyl against other substrate groups. Q140M-T520D, L501H, and V258T-T520D each demonstrated a different two out of these three structural changes: Q140M-T520D did not weaken the hydrogen bond between Q136 and NADPH, L501H did not modify the conformation of E319, and V258T-T520D did not increase the frequency with which eclipsed conformations were adopted by the migrating methyl (Table 2). Interestingly, the UMAP embedding of the more-active-than-WT mutants (Figure 8) partially reflected the structural changes observed for each mutant. Specifically, V258T-T520D overlapped with T520D-L199H in the UMAP projection (Figure 8), and these two variants both had reduced Q136-NADPH hydrogen bonds and increased rotated E319 conformations, but they did not show increased eclipsing of C_5_ in the substrate (Table 2). L501H overlapped with M472Q in the UMAP projection, and these two variants had reduced Q136-NADPH hydrogen bonds and increased eclipsing of the substrate but did not exhibit an increase in rotated E319 conformations (Table 2). Q140M-T520D, the fastest variant, overlapped mostly with T520D and partly with S487A and A497S in the UMAP projection (Figure 8). These four variants behaved differently. Neither Q140M-T520D nor T520D weakened the hydrogen bond between Q136 and NADPH, but they both exhibited increased sampling of rotated E319 and eclipsed C_5_ conformations (Table 2). In contrast, S487A decreased the Q136-NADPH hydrogen bond and significantly increased the frequency of eclipsed substrate conformations but did not increase rotated E319 conformations (Table 2). A497S was the only variant we analyzed for which all three reaction-promoting structural changes were present (Table 2).

### Fast Mutants’ Rate Enhancements Were Primarily Driven by Increased Reaction Probabilities

As was originally done for WT, we used kinetic and equilibrium methods to characterize each of the fast mutants’ reaction dynamics and profile. Here, umbrella sampling with WHAM (an equilibrium method) was used to calculate detailed PMF curves (see Methods) and estimate barrier heights (Δ*G*^‡^) along the reaction order parameter, λ, and TIS-based rate calculations (a kinetic method) were used to decompose the overall rate constants into several terms: (i) the flux factor, which describes the rate at which reactions are attempted, (ii) the probability that an attempt leaving the reactant reaches at least the bottleneck at λ = −0.4 Å (*P*(λ = −0.4 Å|λ_A_)), and (iii) the probability that an attempt that has reached λ = −0.4 Å goes on to reach the product well (*P*(λ_B_|λ = −0.4 Å)).

The PMF curves indicated lower barrier heights, Δ*G*^‡^, for five of the eight mutants relative to WT (*p* ≤ 0.037, one-tailed *t*-test), with the remaining mutants having barrier heights that were comparable to or greater than WT’s (*p* ≥ 0.222). Thus, although a lower barrier along λ was often associated with increased catalytic efficiency, it was not strictly required.

**Figure 13:**
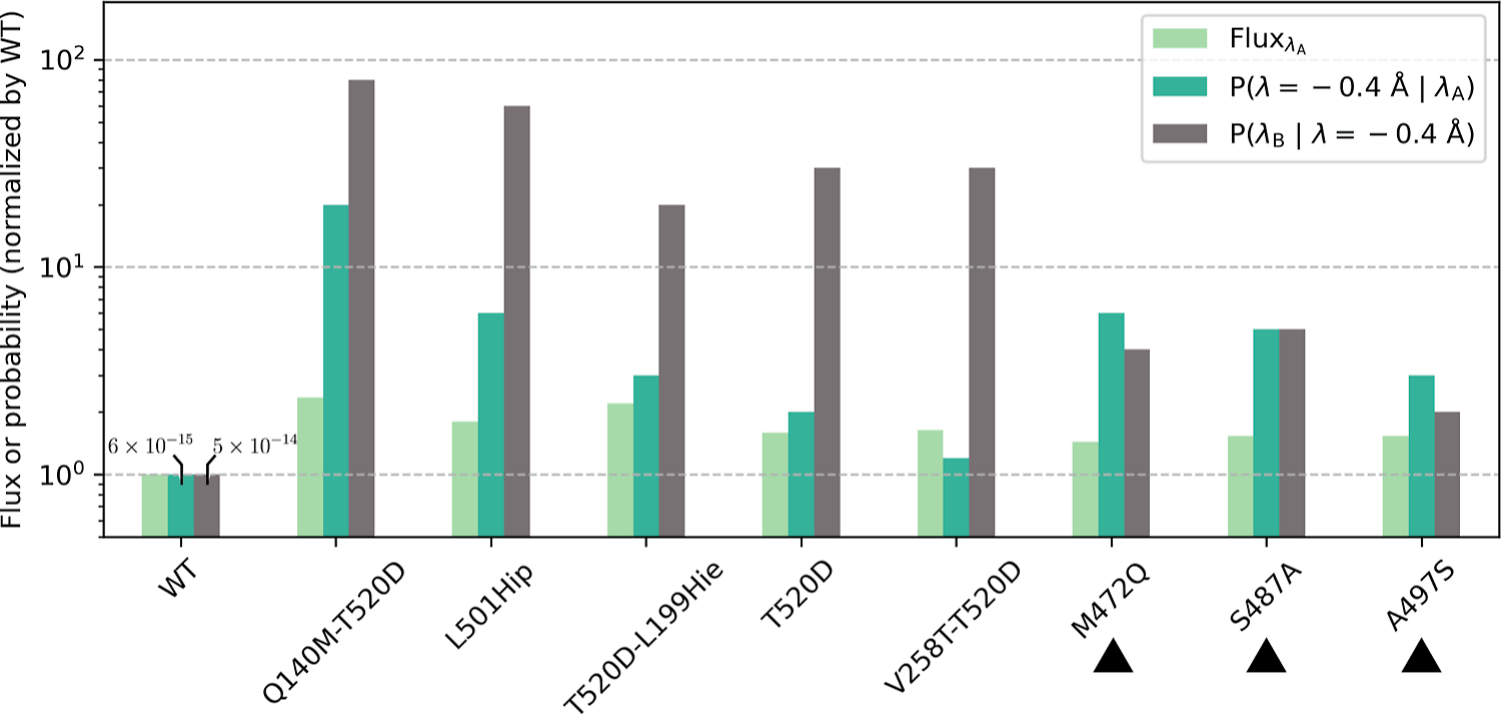
TIS rate terms for WT and the eight high-confidence fast mutants. Flux_λA_ refers to the TIS flux factor, which is the rate at which the enzyme–substrate complex leaves the reactant well. *P*(λ = −0.4 Å|λ_A_) is the probability of reaching the bottleneck at λ = −0.4 Å given an attempt starting from inside the reactant well. *P*(λ_B_|λ = −0.4 Å) is the probability of reaching the product well given an attempt has reached the bottleneck. Black triangles indicate mutants with reaction barrier heights comparable to or greater than WT’s as computed from detailed PMF calculations (see “detailed mode” in Potential of Mean Force Calculations in Methods). Values were averaged from *n* = 9 independent TIS rate calculations and were normalized by WT. The values of the probability terms are explicitly indicated for WT. Hie indicates a neutral histidine residue with the proton in the ϵ position. Hip indicates a doubly protonated, charged histidine residue.

An analysis of the TIS terms led to a number of insights (Figure 13). First, all eight high-confidence fast mutants showed small increases in flux factor (ranging from 1.44- to 2.34-fold) and increases in both of the transition probabilities (ranging from 1.2- to 80-fold) relative to those of WT. The data show, for all eight high-confidence fast mutants, that the increased rate constants were primarily driven by increases in transition probabilities as opposed to flux factors. That is, the mutants’ increased activities were enabled primarily by improvements in gaining and maintaining reaction progress and only somewhat by attempting reactions more frequently.

The terms *P*(λ = −0.4 Å|λ_A_) and *P*(λ_B_|λ = −0.4 Å) describe the transition from the reactant to the product. Taken together, these two terms quantify the degree to which an increase in the overall transition probability was driven by improvements in the initial part of the reaction leading up to the bottleneck versus improvements in the later part of the reaction following the bottleneck. Interestingly, five of the eight fast mutants showed much larger relative improvements in *P*(λ_B_|λ = −0.4 Å) than in *P*(λ = −0.4 Å|λ_A_), with relative differences between these two normalized terms ranging from fourfold in Q140M-T520D to 25-fold in V258T-T520D. This suggested that their increased catalytic efficiencies were especially pronounced along the profile of the reaction following the bottleneck as opposed to before. These five mutants all also had lower computed Δ*G*^‡^ than WT. In contrast, the three mutants with Δ*G*^‡^ comparable to WT showed relative increases to *P*(λ_B_|λ = −0.4 Å) and *P*(λ = −0.4 Å|λ_A_) of similar size, with the relative differences here ranging from only 0.7-fold in M472Q and A497S to 1-fold in S487A. Therefore, these three mutants’ increased activities were enabled by roughly equal improvements to reaction kinetics before and after the reaction bottleneck (Figure 13). It is interesting that while M472Q, S487A, and A497S all had computed free energy barriers comparable to WT's, they each still showed relative improvement in all three of the kinetic TIS terms (Figure 13). Collectively, these data suggest that barrier height is but one contribution to enzyme specific activity (*k*_cat_), and that some of the other contributions are inherently kinetic in nature.

## Conclusions

This work presents a computational test of an enzyme engineering strategy that we previously proposed.^9^ Namely, the introduction of specific mutations designed to stabilize and thus increase the probability of reactive-like conformations could be an effective means to increase enzyme specific activity (*k*_cat_). In studying the rate-limiting step for the enzyme KARI, we observed that the flux of reaction attempts dropped by 20 orders of magnitude between leaving the reactant basin and arriving at the product well. The vast majority of trajectories were turned back, and such trajectories had a reasonable probability of occurring throughout the climb toward the TS region, which suggested that there were ample opportunities for improvement but also that mutants could be required to exert effects across a large stretch of the reaction profile.

We introduced a pipeline that used protein redesign methodology to implement the design objective of identifying mutants that differentially stabilized reactive-like over nonreactive-like conformations relative to WT. Because even mutants that successfully achieved the objective could introduce detrimental effects particularly after the reactive complex left the reactant well, the pipeline included additional characterizations of both the relative populations of reactive-like conformations as well as of the estimated changes in barrier heights. Of 54 mutants selected from this process, 28 were computed by TIS to have increased *k*_cat_ relative to WT. Our study focused on 8 of these 28 which were shown to be significant by a thorough statistical test. They ranged in their improvements from a low of sevenfold to a high of 20,000-fold. These rigorous computational tests established the feasibility of the engineering approach put forth in this study and thereby validated the underlying design hypothesis that stabilization of reactive-like complexes can lead to enhanced specific activity. Experimental testing, which should be done in the future to verify designed mutants’ real-world applicability, will speak toward the accuracy of the simulation setup used in this study but not necessarily the validity or feasibility of the design hypothesis embodied by the design approach put forth here. That is, experimental testing of mutants may uncover disparities between measured and calculated enzyme activities, but these differences may be due to factors beyond the scope of this work’s focus on the feasibility of enhancing reactive-like conformations for enhancing enzymatic activity. The most direct and clear test of the design hypothesis put forth in this work is a rate constant calculation of the isomerization reaction in the context of the simulation setup used to guide the selection of the candidate mutants, which was reported in this study.

Mechanistic characterization of the eight high-confidence fast mutants identified three key structural changes shared by multiple mutants involving the substrate conformation (migrating methyl eclipse), the electrostatic environment of the substrate–coordinating Mg^2+^ ions (E319 conformational shift), and the orientation of an NADPH-coordinating side chain (Q136). The latter two structural changes were markers that tended to correlate with other nearby structural changes that also tended to be associated with reactivity. The analysis showed that the mutants increased activity essentially by concentrating conformational traits associated with reactivity in the active site of the WT enzyme. That is, for these mutants, the evidence suggested that their enhanced activity arose from more of the same features that made the WT itself reactive. It is possible that additional structural mechanisms different from those active in WT reactivity also contributed, but none were identified. While we found three structural changes associated with increased reactivity, most mutants exhibited only two of the three. This suggests a model in which there are multiple supporting interactions, perhaps corresponding to different reaction channels, that can enhance the reaction rate, and only some of these are required to drive a positive net change in activity. The analyses further showed that the mutants exerted their effects across much of the course of the reaction from leaving the reactant basin through passing the TS region by essentially creating the conditions under which the reaction had a reduced probability of being turned back throughout much of its profile.

While our structural analysis focused on mutants in the neighborhood of the active site, roughly corresponding to the second or third solvation shell of the substrate, we also identified beneficial mutations much farther from the active site. These distal mutants similarly increased the population of reactive-like conformations. However, given their separation from the active site, it was difficult to ascertain how the mutations led to increased reactive-like conformational sampling. Collectively, these results show that prereaction enzyme–substrate conformations are related to catalytic efficiency, and enzyme redesign toward increased specific activity can be facilitated by selecting mutations that more frequently populate reactive-like conformations. In future work, it would be interesting to determine whether experimentally selected mutations also tend to be effective through creating a surplus of WT-like reactive conformations.

## Methods

### Structure Preparation and Equilibration

A crystal structure of WT KARI (from *S. oleracea*) bound to TS analogue (*N*-hydroxy-*N*-isopropyloxamate), NADPH, and Mg^2+^ ions was retrieved from the Protein Data Bank (accession code 1YVE^47,60^) and prepared as described by Bonk^9^ and Silver^48^ with the exception that here both chains of the homodimer were kept. Substrate O_6_ was deprotonated and E496 protonated, in line with previous studies suggesting that this was the reactant state immediately prior to the methyl migration (isomerization reaction).^61^ The enzyme–substrate complex was energetically minimized using a hybrid QM/MM force field in CHARMM^62,63^ compiled with SQUANTUM (see the Simulation Methodology section). Prior to production molecular dynamics simulations, the minimized enzyme–substrate structure was subjected to a 200 ps equilibration run as described in the Simulation Methodology section. The structure at the end of this simulation was used for downstream analyses and for initiating production simulations.

### Simulation Methodology

A custom modified implementation of CHARMM^62,63^ developmental version 39a1 with SQUANTUM was used for running all enzyme–substrate energy minimizations and molecular dynamics simulations.^50^ CHARMM’s SQUANTUM QM/MM implementation was used to treat the QM region with the semiempirical AM1^64^ quantum mechanical force field. This region included substrate, Mg^2+^ ions, and coordinating waters, the nicotinamide group of NADPH, and the side chains of residues D315, E319, and E496. Parameters for Mg^2+^ were taken from previous work by Stewart.^65^ Atoms outside of the QM region were treated with CHARMM36’s all-atom force field.^66^ The Generalized Hybrid Orbital method^67^ was used to treat the QM/MM boundary atoms: the alpha carbons of residues D315, E319, and E496 as well as the C_5_^’^ atom of the ribose ring in NADPH, linking to the nicotinamide group. All molecular dynamics simulations were performed in vacuo with a distance dependent dielectric (1*r*). Temperature was controlled near 300 K using Langevin dynamics with a friction coefficient (FBETA) of 1 ps^–1^. All simulations used a 1 fs integration time step.

### Potential of Mean Force Calculations

Umbrella sampling with the weighted histogram analysis method^68^ (WHAM) was used for all potential of mean force (PMF) calculations. The umbrella sampling was performed using the RXNCOR module to apply umbrella bias terms, and WHAM was performed using software from Grossfield.^69^ To calculate PMF curves along the order parameter λ, which was defined as the difference between the lengths of the substrate’s breaking bond (C_4_–C_5_) and the substrate’s forming bond (C_5_–C_7_) in units of Å, umbrella simulations were implemented using a force constant of 200.0 kcal/(mol·Å^2^) to harmonically constrain λ at umbrella term bias minima spanning λ = −1.2 Å to λ = 1.2 Å. For −0.5 Å < λ < 0.5 Å, consecutive umbrella windows were spaced 0.0325 Å apart; for λ outside this range, consecutive windows were spaced 0.0975 Å apart. Each umbrella simulation was started from a structure that was prepared and equilibrated as described above. Umbrella simulations were run in either of two modes. In screening mode, rapid calculations were performed to help characterize proposed mutants and choose which ones should be studied by TIS and other methods. Screening simulations were run for 5 ps with no equilibration. In the detailed mode, more resource-intensive simulations were run to obtain an accurate PMF calculation. Detailed PMF curves along λ were constructed from 100 ps umbrella simulations, with the first 50 ps reserved for equilibration. Three statistical replicates were obtained for each detailed PMF curve.

### Seed Trajectory Generation

Initial reactive trajectories [i.e., pathways connecting the reactant well (state A, λ < – 0.8 Å) to the product well (state B, λ > 0.8 Å)] were found by randomly sampling TS-like enzyme–substrate conformations from umbrella sampling simulations centered near λ ≈ 0 Å, removing their constraints, and executing TIS shooting moves starting from them (see below). If the reconstructed pathway from the forward and backward integrations connected the reactant and product states, then the trajectory was selected as a successful starting seed trajectory. Each trajectory was then equilibrated for 2000 additional TIS shooting moves.

### TIS Shooting Moves

TIS involves collecting new dynamic pathways by using a Monte Carlo sampling strategy. At each sampling iteration, a new pathway is attempted from the current one using a shooting move.^41,70−72^ We followed the TIS algorithm described by van Erp et al.^41^ Within this framework, we implemented shooting moves following the suggested procedures outlined by Dellago et al.^70^ in Selecting Phase-Space Displacements with the addition of sampling perturbations to kinetic energy using the protocol described by Geissler and Chandler,^71^ which we found necessary in order to independently control the sizes of momenta displacements and kinetic energy changes. The TIS method and its shooting move procedure were implemented using a custom Python wrapper around CHARMM 39a1; CHARMM was used only for the individual dynamics simulations.

### TIS Rate Constant Computations

TIS rate constant calculations were performed in accordance with the theory and procedures outlined by van Erp et al.^41^ The calculation involved computing two terms: the effective flux factor Φ_*A*_ and the probability factor *P*(λ_B_|λ_A_). To determine the flux factor, 30 independent 400 ps QM/MM simulations of reactant well dynamics were performed. The probability factor, *P*(λ_B_|λ_A_), was calculated as the product of a series of conditional probability terms, where each term reported on the probability of reaching λ_*i*+1_ having reached λ_*i*_, such that *P*(λ_B_|λ_A_) = *P*(λ_B_|λ_*n*_)∏_*i*=1_^*n*–1^*P*(λ_*i*+1_|λ_*i*_). Here, λ_B_ indicates the interface at the edge of the product well, and λ_A_ = λ_1_ indicates the interface at the edge of the reactant well. A total of 29 *P*(λ_*i*+1_|λ_*i*_) interface ensembles was sampled for *i* from −0.8 to 0 Å. For each ensemble, 6000 shooting moves (described above) were attempted with the first 3000 being reserved for equilibration and not counted toward the path ensemble. We repeated this procedure for three statistical replicates for each of three seed trajectories, giving nine independent estimates of *k*_cat_ for each enzyme variant analyzed. The rate calculation procedure and the execution of its TIS simulations were handled using a custom Python wrapper around CHARMM; CHARMM was used for running only the individual dynamics simulations.

### Machine Learning

For reactive pathways and nearly reactive pathways that reached λ > – 0.4 Å, 20 independent pathway ensembles were sampled across 10 unique starting seed trajectories, and 70 structural features (interatomic distances, angles, and torsions) describing the active site were calculated at each time step (Table S1). These features included the 68 described by Bonk et al. with the addition of two dihedral angles describing the conformation of the substrate: (i) the minimum dihedral angle across atoms O_6_, C_4_, C_5_, and any one of the hydrogens bound to C_5_ and (ii) the minimum dihedral angle across atoms O_8_, C_7_, C_5_, and any one of the hydrogens bound to C_5_. Trajectories were time-aligned by defining *t* = 0 fs at the trough of the last compression of the breaking bond as done by Bonk et al. Single time points were sampled from each trajectory within some 30 fs time window and weighted in accordance with their pathway count from TIS.

LR models were trained on all 70 features and with LASSO regression^73^ to select optimal subsets of 5, 10, or 20 features. Models were then retrained, without regularization, using only the feature subset to report its performance. NN models were constructed with one hidden layer with 70 nodes and ReLU activation (this architecture consistently had the most favorable Bayesian information criterion (BIC)^74,75^ scores, and we saw limited predictive performance improvement when increasing the number of hidden layers or nodes) and trained using a learning rate of 0.001, α = 0.0001, and batch size of 200. These choices were supported by a grid search over the hyperparameter values. In addition to training on all 70 features, greedy sequential feature selection was used to choose high-performing subsets with only 5, 10, or 20 features, on which NNs were evaluated. LR and NN models were trained on data from different 30 fs time windows spanning −200 to 0 fs. Performance metrics are reported for held-out data using 5-fold cross validation.

### Protein Redesign

We implemented a protein redesign procedure to find mutations that energetically stabilized reactive-like conformations relative to nonreactive-like ones, based on WT. Subsets of representative reactive-like and nonreactive-like structures were selected by LR and NN models and used to construct the objective function in the OSPREY 3.0^58^ implementation of the COMETS algorithm^57^ for multistate DEE/A*-based protein redesign. In brief, the algorithm identified new side chain identities that minimized the energetic design objective defined over the selected reactive-like and nonreactive-like structures. We defined the design objective *f*, a function of sequence *s*, as  where *N*_R_ and *N*_NR_ are respectively the numbers of reactive and nonreactive structures (three), *E*_R,*i*_ is the potential energy of the *i*th reactive structure, and *E*_NR,*i*_ is the potential energy of the *i*th nonreactive structure. The design objective score for each mutant *s* was reported as the difference between *f*(s) and the corresponding objective score for the WT sequence, *f*(WT). Stability constraints were enforced such that the designed mutations’ energetically optimized reactive structures were no more than 5 kcal/mol less stable than the starting sequence’s optimized reactive structures; otherwise, the mutation was disallowed. Mutants that were selected for further characterization and screening with QM/MM simulations were first equilibrated for 200 ps.

### Mutant Characterization and Screening

Following protein redesign rounds, each candidate mutant was evaluated by its objective score, its dynamics in the reactant well, and its approximate reaction energy barrier. The objective score was computed as described in Protein Redesign. Reactant well dynamics were tracked by running 30 independent 400 ps QM/MM dynamics simulations in the reactant well and computing the fraction of time steps that were on the reactive side of the machine learning classifiers (i.e., sampling reactive-like conformations). Reaction barrier height (Δ*G*^‡^) was estimated using an approximate PMF calculation (see “screening mode” in the Potential of Mean Force Calculations section). Candidate mutants were prioritized for more expensive TIS *k*_cat_ calculations based on these three metrics.

### Tracking Reactive-Like Structural Characteristics

Statistics were tracked for three reactive-like structural characteristics that were observed across multiple mutants with significantly increased calculated activity relative to WT: loss of Q136-NADPH hydrogen bonds, rotated E319 side chains, and eclipsed C_5_ conformations. We defined several criteria to track when these characteristics were present. We considered the Q136-NADPH hydrogen bond to be present only if the distance from Q136’s donated proton to the amide carbonyl of NADPH (the acceptor) was less than 2.3 Å, the angle between the axis from Q136’s amine nitrogen to its donated proton and the purported hydrogen bond was within 30° of 180°, and the angle between the purported hydrogen bond and NADPH’s amide carbonyl group was within 30° of 120°. If any of these three conditions were not met, then the conformation was considered to have no Q136-NADPH hydrogen bond. The rotated E319 conformation was recorded as present for conformations in which the angle between the axis joining E319’s carboxylate oxygens and the axis joining atoms C_4_ and O_6_ in the substrate was less than 75°. An eclipsed C_5_ conformation was reported if the dihedral angle measured between any one of the hydrogens bonded to C_5_ and either O_6_ or O_8_ was less than 30°.

### Statistics

Statistics were performed using SciPy in Python. Values are reported as the average ± the standard error of the mean (SEM). Normality of the data was evaluated using an *F*-test. Equality of variance was evaluated using the Shapiro–Wilk test. For normal data with equal variance, comparisons between two groups were performed using a *t*-test. For data that were not normal or had unequal variance, comparisons between two groups were performed using a Mann–Whitney *U* test. Specifically, for the comparison of mutant and WT *k*_cat_ values calculated with TIS, a one-sided Mann–Whitney *U* test was used (the alternative hypothesis being that the mutant value was larger than the WT one), and significance was detected using the Benjamini–Hochberg procedure to control the false discovery rate (FDR) at α = 0.05.

## Supporting Information Available

Additional tables, figures, and details pertaining to computational methods and simulation setup (PDF)
