# Supplementary material for: Substrate Turnover Dynamics Guide Ketol-Acid Reductoisomerase Redesign for Increased Specific Activity
Source: ACS Catal. 2024 Jun 26;14(14):10491–509. doi: 10.1021/acscatal.4c01446 (PMC11264209; doi:10.1021/acscatal.4c01446)
Supplement: Supplementary file 1 — cs4c01446_si_001.pdf [file cs4c01446_si_001.pdf]

Substrate turnover dynamics guide ketol-acid reductoisomerase redesign for increased specific activity (Supporting Information)

Elijah Karvelis,[†,‡] Chloe Swanson,[†,‡] and Bruce Tidor[*,†,‡,¶]

†*Department of Biological Engineering, Massachusetts Institute of Technology, Cambridge, MA 02139, USA*

‡*Computer Science and Artificial Intelligence Laboratory, Massachusetts Institute of Technology, Cambridge, MA 02139, USA*

¶*Department of Electrical Engineering and Computer Science, Massachusetts Institute of Technology, Cambridge, MA 02139, USA*

E-mail: tidor@mit.edu

Supporting Figures and Tables

Table S1: The 70 structural features describing the active site.[a]

Distance AC6/O2,NDP/N7N	Distance NDP/H4N2,NDP/C4N
Distance AC6/O2,NDP/O7N	Distance NDP/N7N,NDP/O2N
Distance AC6/O3,MG6/H24	Angle NDP/C4N,NDP/N1N,NDP/C1NQ
Distance AC6/O6,MG6/M16	Angle NDP/C6N,NDP/C3N,NDP/C7N
Distance AC6/O8,GLU496/HE2	Angle NDP/N7N,NDP/H72N,NDP/O2N
Distance AC6/O8,MG6/M17	Dihedral NDP/C2N,NDP/C3N,NDP/C7N,NDP/N7N
Distance GLU319/OE1,AC6/C5	Dihedral NDP/C2NQ,NDP/C1Nq,NDP/N1N,NDP/C6N
Distance MG6/H25,AC6/O6	Dihedral NDP/C4N,NDP/C3N,NDP/C7N,NDP/O7N
Distance MG6/H26,AC6/O6	Dihedral NDP/H1NQ,NDP/C1NQ,NDP/N1N,NDP/C2N
Distance MG6/H27,AC6/O6	Distance MG6/O18,MG6/M17
Distance MG6/H28,AC6/O6	Distance MG6/O19,MG6/M17
Distance MG6/H31,AC6/O6	Distance MG6/O20,MG6/M17
Distance MG6/H32,AC6/O6	Distance MG6/O21,MG6/M16
Distance MG6/M16,AC6/O3	Distance MG6/O22,MG6/M16
Distance MG6/M17,AC6/O6	Angle MG6/H23,MG6/O22,MG6/M16
Distance NDP/H4N2,AC6/C4	Angle MG6/H24,MG6/O22,MG6/M16
Angle AC6/O6,MG6/M16,AC6/O3	Angle MG6/H25,MG6/O21,MG6/M16
Angle AC6/O8,MG6/M17,AC6/O6	Angle MG6/H26,MG6/O21,MG6/M16
Angle MG6/M17,AC6/O6,MG6/M16	Angle MG6/H27,MG6/O20,MG6/M17
Distance AC6/C1,AC6/C4	Angle MG6/H28,MG6/O20,MG6/M17
Distance AC6/C1,AC6/O2	Angle MG6/H29,MG6/O18,MG6/M17
Distance AC6/C1,AC6/O3	Angle MG6/H30,MG6/O18,MG6/M17
Distance AC6/C4,AC6/C7	Angle MG6/H31,MG6/O19,MG6/M17
Distance AC6/C4,AC6/O6	Angle MG6/H32,MG6/O19,MG6/M17
Distance AC6/C5,AC6/C4	Distance GLU496/OE2,GLU496/HE2
Distance AC6/C5,AC6/C7	Distance GLN136/NE2,NDP/O7N
Distance AC6/C7,AC6/C9	Distance MG6/H25,MG6/O21
Distance AC6/C7,AC6/O8	Distance MG6/H26,MG6/O21
Angle AC6/C1,AC6/C4,AC6/C7	Distance MG6/H27,MG6/O20
Angle AC6/C4,AC6/C7,AC6/C5	Distance MG6/H28,MG6/O20
Angle AC6/C4,AC6/C7,AC6/C9	Distance MG6/H31,MG6/O19
Angle AC6/C5,AC6/C4,AC6/C1	Distance MG6/H32,MG6/O19
Angle AC6/C5,AC6/C7,AC6/C9	Angle GLN136/NE2,GLN136/HE22,NDP/O7N
Dihedral AC6/C1,AC6/C5,AC6/C7,AC6/C4	Dihedral AC6/O6,AC6/C4,AC6/C5,AC6/C5-H
Dihedral AC6/C5,AC6/C4,AC6/C7,AC6/C9	Dihedral AC6/O8,AC6/C7,AC6/C5,AC6/C5-H

[a] AC6, substrate. NDP, NADPH.

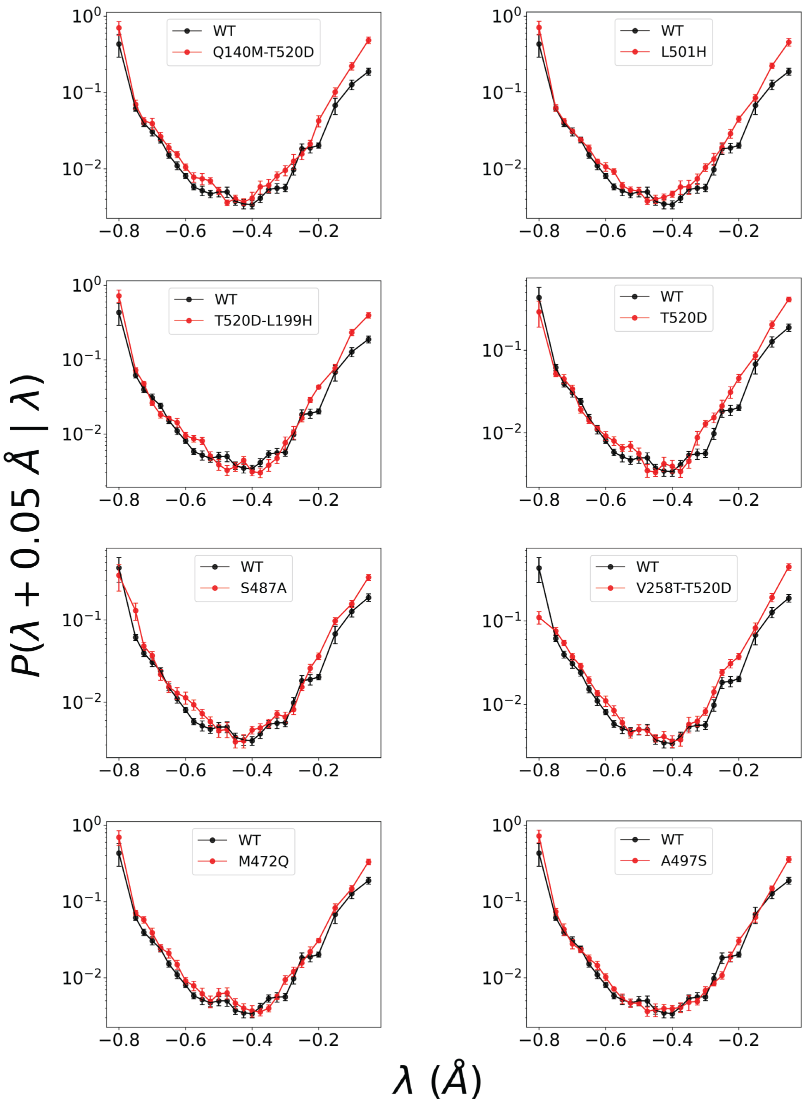

Figure S1: Kinetic profile comparison between WT and the eight mutants with significantly increased computed k_{cat}. Each plot indicates the probability of an attempted reaction making incrementally further progress along λ given that a certain level of progress was already made ($P(\lambda + 0.05\ \text{Å} \mid \lambda)$). Circle markers indicate the average and error bars designate $\pm$ SEM (n = 9 independent TIS rate calculations).

Table S2: LR model features and coefficients (trained on data from −160 to −130 fs).[a]

Feature	LR Coefficient (β_i)
Distance AC6/O6,MG6/M16	0.083875
Distance AC6/O8,MG6/M17	−0.062094
Distance GLU319/OE1,AC6/C5	0.051582
Angle AC6/O6,MG6/M16,AC6/O3	−0.037827
Angle MG6/M17,AC6/O6,MG6/M16	−0.187901
Distance AC6/C5,AC6/C7	0.061126
Angle AC6/C1,AC6/C4,AC6/C7	0.002063
Dihedral NDP/C2N,NDP/C3N,NDP/C7N,NDP/N7N	−0.067934
Distance GLN136/NE2,NDP/O7N	0.041946
Angle GLN136/NE2,GLN136/HE22,NDP/O7N	−0.037056

[a] AC6, substrate. NDP, NADPH.

Supporting Methods

Structure Preparation and Equilibration

A crystal structure of WT KARI (from *Spinacia oleracea*) bound to transition state analogue (N-hydroxy-N-isopropyloxamate), NADPH, and Mg^{2+} ions was retrieved from the Protein Data Bank (accession code 1YVE[1,2]) and prepared similar to as described by Bonk[3] and Silver[4] with the exception that here both chains of the homodimer (I and J) were kept. While the mature KARI monomer starts with M72, the crystallographers reported disordered N-termini, and the first residues with defined positions in chains I and J were A83 and F86, respectively.[1] CHARMM[5,6] was used to build residues A83, T84, and T85 onto the N-terminus of chain J. Both of the N-termini were then capped with an acetyl group. Consistent with their environment, all histidine residues were kept neutrally protonated in both chains: 103-δ, 215-δ, 226-δ, 232-δ, 280-ϵ, 328-ϵ, 484-δ, 506-ϵ, 564-ϵ. Crystallographic waters outside the active site were removed if they made fewer than three hydrogen bonds. Substrate O_6 was deprotonated and E496 protonated, in line with previous studies suggesting that this was the reactant state immediately prior to the methyl migration (isomerization reaction).[7] Specifically, protonated E496 could donate a hydrogen bond to the substrate O_8 carbonyl, which was previously found to play a critical role in transition and product state stabilization.[7]

The geometry of the substrate 2(S)-acetolactate, Mg^{2+} ions, and Mg^{2+}-coordinating waters was found using ground state energy minimization implemented with GAUSSIAN03[8] at the rhf/6-31g* level of QM theory. To dock this structure into the enzyme active site, the substrate was aligned to the transition state analogue. The enzyme-substrate complex was then energetically minimized in two stages using a hybrid QM/MM force field in CHARMM[5,6] compiled with SQUANTUM (see Simulation Methodology). First, all atoms were held fixed except for hydrogens and the first three residues on chain J, and the structure was then subjected to 100 steps of steepest descent minimization followed by 100 steps of adopted basis Newton-Raphson minimization. In the second minimization stage, the substrate, Mg^{2+} ions, and coordinating oxygen atoms on D315, E319, and E496 were held fixed while all other atoms within 8 Å of the substrate were harmonically constrained with a force constant of 50 $\mathrm{kcal/(mol \cdot Å^2)}$. The structure then underwent 10 rounds comprised of 100 steps of steepest descent minimization followed by 100 steps of adopted basis Newton-Raphson minimization. The harmonic constraints were reset between each round. Prior to production molecular dynamics simulations, the minimized enzyme-substrate structure was subjected to a 200-ps equilibration run as described in Simulation Methodology. The structure at the end of this simulation was used for downstream analyses and for initiating production simulations.

Simulation Methodology

A custom modified implementation of CHARMM[5,6] developmental version 39a1 with SQUANTUM was used for running all enzyme-substrate energy minimizations and molecular dynamics simulations. This implementation contained a custom modified RXNCOR module for running TIS simulations.[9] CHARMM's SQUANTUM QM/MM implementation was used to treat the QM region with the semiempirical AM1[10] quantum mechanical force field. This region included substrate, Mg^{2+} ions and coordinating waters, the nicotinamide group of NADPH, and the side chains of residues D315, E319, and E496. Parameters for Mg^{2+} were taken from previous work by Stewart.[11] Atoms outside of the QM region were treated with

CHARMM36's all-atom force field.[12] The Generalized Hybrid Orbital method[13] was used to treat the QM/MM boundary atoms: the alpha carbons of residues D315, E319, and E496 as well as the C_5' atom of the ribose ring in NADPH, linking to the nicotinamide group. All molecular dynamics simulations were performed *in vacuo* with a distance dependent dielectric ($1r$). Temperature was controlled near 300 K using Langevin dynamics with a friction coefficient ($FBETA$) of 1 ps^{-1}. All simulations used a 1-fs integration time step.

Potential of Mean Force Calculations

Umbrella sampling with the weighted histogram analysis method[14] (WHAM) was used for all potential of mean force (PMF) calculations. The umbrella sampling was performed with modified CHARMM version 39a1 using the RXNCOR module to apply umbrella bias terms, and WHAM was performed using software from Grossfield.[15]

To calculate PMF curves along the order parameter λ, which was defined as the difference between the lengths of the substrate's breaking bond ($C_4 - C_5$) and the substrate's forming bond ($C_5 - C_7$) in units of Å, umbrella simulations were implemented using a force constant of 200.0 kcal/(mol $\cdot$ Å^2) to harmonically constrain λ at umbrella term bias minima spanning $\lambda = -1.2$ Å to $\lambda = 1.2$ Å. For -0.5 Å $< \lambda <$ 0.5 Å, consecutive umbrella windows were spaced 0.0325 Å apart; for λ outside this range, consecutive windows were spaced 0.0975 Å apart. Each umbrella simulation was started from a structure that was prepared and equilibrated as described above.

Umbrella simulations were run in either of two modes. In screening mode, rapid calculations were performed to help characterize proposed mutants and choose which ones should be studied by TIS and other methods. Screening simulations were run for 5 ps with no equilibration. In detailed mode, more resource-intensive simulations were run to obtain an accurate PMF calculation. Detailed PMF curves along λ were constructed from umbrella simulations that were run for 100 ps, with the first 50 ps reserved for equilibration.

Seed Trajectory Generation

Initial reactive trajectories (i.e., pathways connecting the reactant well (state A, $\lambda < -0.8$ Å) to the product well (state B, $\lambda > 0.8$ Å)) were found by randomly sampling TS-like enzyme-substrate conformations from umbrella sampling simulations centered near $\lambda \approx 0$ Å, removing their constraints, and executing TIS shooting moves starting from them (see below). If the reconstructed pathway from the forward and backward integrations connected the reactant and product states, then the trajectory was selected as a successful starting seed trajectory. Each trajectory was then equilibrated for 2,000 additional TIS shooting moves. For this procedure, each initially generated seed trajectory was used to start sampling a TIS ensemble comprised of 2,000 shooting moves, and the latest trajectory to be accepted into this ensemble was labeled the equilibrated seed path and used for initializing all downstream TIS simulations that generated pathway ensembles for production data.

TIS Rate Constant Computations

TIS rate constant calculations were performed in accordance with the theory and procedures outlined by van Erp *et al.*[16] The calculation involves computing two terms: the effective flux factor Φ_A and the probability factor $P(\lambda_B \mid \lambda_A)$. The flux factor describes the frequency with which the enzyme-substrate complex exits the reactant well, and the probability factor reflects the likelihood that the enzyme-substrate complex will reach the product well given that it has exited the reactant well.

To determine the flux factor, 10 independent 400-ps QM/MM simulations were performed starting from each of three reactant structures, where each reactant structure was derived from a different seed trajectory. Therefore, 30 independent simulations were performed in total. In each simulation, the flux was computed as the number of times the the trajectory crossed λ_A, having come from inside the reactant well, divided by the total amount of time that the trajectory spent inside the reactant well (i.e., total time where $\lambda < \lambda_A$). This value was reported for each seed trajectory as the average across its 10 independent simulations.

The probability factor is generally a very small value. To achieve reasonable statistics, the TIS procedure breaks $P(\lambda_B \mid \lambda_A)$ up into the product of a series of conditional probability terms, where each term reports on the probability of reaching λ_{i+1} having reached λ_i, such that $P(\lambda_B \mid \lambda_A) = \prod_{i=1}^{n-1} P(\lambda_{i+1} \mid \lambda_i) P(\lambda_B \mid \lambda_n)$ and each term can be efficiently computed from a TIS ensemble. Here, λ_B indicates the interface at the edge of the product well, and $\lambda_A = \lambda_1$ indicates the interface at the edge of the reactant well. A total of 29 $P(\lambda_{i+1} \mid \lambda_i)$ interface ensembles were sampled for i from -0.8 Å to 0 Å, in triplicate, starting from each of the three unique seed trajectories. To ensure adequate sampling, the interfaces between $\lambda = -0.75$ and $\lambda = -0.15$ were spaced 0.025 Å apart, and the remaining interfaces from $\lambda = -0.8$ to $\lambda = -0.75$ and from $\lambda = -0.15$ to $\lambda = 0$ were spaced 0.05 Å apart. For each ensemble, a total of 6,000 shooting moves (described below) were attempted. The first 3,000 moves were reserved for equilibration and only the last 3,000 shots were used in downstream analyses.

This procedure gives three statistical replicates for each of three seed trajectories, or nine independent evaluations of $P(\lambda_B \mid \lambda_A)$ in total. Each estimated $P(\lambda_B \mid \lambda_A)$ was multiplied by its corresponding seed trajectory's average Φ_A to give an estimate of k_{cat}. These nine k_{cat} values were treated as independent.

The rate calculation procedure and the execution of its TIS simulations were handled using a custom Python wrapper around CHARMM; CHARMM was only used for running the individual dynamics simulations.

TIS Shooting Moves

TIS involves collecting new dynamical pathways using a Monte Carlo sampling strategy. At each sampling iteration, a new pathway is attempted from the current one using a shooting move.[16–19] The general shooting move procedure involves selecting a frame from the current pathway and initiating new pathway simulations from it. To ensure that the new pathway simulations do not replicate the old (current) pathway, the selected frame's momenta must be

perturbed unless Langevin dynamics are used. While all of our QM/MM simulations used Langevin dynamics with a friction coefficient of 1 ps^{-1} to control temperature, we found that the random displacements from this setup were insufficient for generating shooting moves that efficiently sampled pathway phase space. Therefore, direct perturbations to selected frames' momenta were applied for all shooting moves, similar to as was described for TIS with deterministic dynamics.[16] To independently control the sizes of the changes to momenta and kinetic energy, we used the two-step procedure for generating shooting move displacements described by Geissler and Chandler,[18] wherein a displacement to the momenta and to the kinetic energy are independently sampled. The (perturbed) momenta are then rescaled to give the new kinetic energy.[18] Net zero linear and angular momenta were maintained at each shooting move following the procedure outlined by Dellago *et al.*[17] The entire shooting move can be summarized as follows. A frame was randomly selected from the current pathway with uniform probability across all time points for which the enzyme-substrate complex hasn't yet returned to state A or reached state $i + 1$, where state $i + 1$ indicates the next interface. Velocity perturbations were sampled for each atom, along each axis, from a Gaussian distribution with $\mu = 0$ and and $\sigma = \sqrt{k_B T/m_a}$ where k_B is the Boltzmann constant, T is temperature, and m_a is the atom's mass. The velocity displacements were then rescaled by a factor of 0.5. The purpose of the scaling factor 0.5 was to control the overall pathway acceptance rate by way of affecting the size of the velocity displacements. A kinetic energy displacement was sampled from a Gaussian distribution with $\mu = 0$ and $\sigma = \sqrt{\frac{3Nk_B^2T^2}{2}}$ where N is the total number of atoms.[17,18] The updated kinetic energy, k_n, was accepted with probability min $\left[1, \mathrm{e}^{(k_BT)^{-1}(k_o-k_n)}(k_n/k_o)^{3N/2-1}\right]$ where k_o is the original kinetic energy before the update.[18] If rejected, then the entire trial was terminated and the old pathway recounted toward the pathway ensemble. If accepted, then after application of the velocity displacements, the net linear and angular momenta were removed,[17] and the momenta were rescaled to give the newly updated kinetic energy.[17,18] The perturbed frame with new momenta and new kinetic energy was then used to initialize

the newly attempted pathway simulation by integrating forward and backward in time (the backward simulation was implemented by negating all momenta) until crossing either the $\lambda = \lambda_A$ interface or the $\lambda = \lambda_{i+1}$ interface. Integration was stopped once either interface was reached using a modified RXNCOR module in CHARMM 39a1.[9] The pathway was rejected if the backward trajectory reached the next interface λ_{i+1} before state A, or if the entire trial pathway failed to cross the current interface λ_i. Otherwise, the trial pathway was accepted with probability $\min[1, L_o/L_n]$ where L_o and L_n are the lengths, in discrete time steps, of the old and new pathways, respectively.[16] This final acceptance criterion is necessary to achieve detailed balance. The TIS method and its shooting move procedure were implemented using a custom Python wrapper around CHARMM 39a1; CHARMM was only used for the individual dynamics simulations.

Machine Learning

For reactive pathways and nearly reactive pathways that reached $\lambda > -0.4$ Å, 20 independent pathway ensembles were sampled across 10 unique starting seed trajectories. Starting seed trajectories were generated, and shooting moves performed, as previously described. However, when generating pathways that were used to train and evaluate ML models, accepted trajectories' dynamics were continued an additional 1,100 fs or 300 fs after crossing the reactant (λ_A) or product (λ_B) interfaces, respectively. The integration of these additional time steps enabled downstream analyses of the dynamics occurring before and after attempted reactions. In a post-processing step, 70 structural features (interatomic distances, angles, and torsions) describing the active site were calculated at each time step for every accepted trajectory. These 70 features included the 68 described by Bonk *et al.* with the addition of two dihedral angles describing the conformation of the substrate: (i) the minimum dihedral angle across atoms O_6, C_4, C_5, and any one of the hydrogens bound to C_5 and (ii) the minimum dihedral angle across atoms O_8, C_7, C_5, and any one of the hydrogens bound to C_5. Different trajectories were time-aligned by defining $t = 0$ at the trough of the

last compression in the breaking bond, as done by Bonk *et al.* This final compression occurs right before the enzyme-substrate complex exits the reactant well, and it corresponds to one of the last time steps when the enzyme-substrate complex is still in the reactant state before attempting to convert to product.[3]

We wanted to train ML models whose performance did not depend on the precise timing of structural features, but rather on characteristics that were generally related to reactivity across multiple time points. Therefore, after pooling all post-processed trajectory data, single time points were sampled from each trajectory within some 30-fs time window. These individual time points (one for each trajectory) were then weighted in accordance with their pathway count from TIS. This weighting step ensured equal representation of the non-reactive and reactive pathways.

LR models were developed using the scikit-learn module in Python. We used 'L1' regularization to apply LASSO regression to find and train on optimal subsets of structural features.[20] By controlling the size of the penalty term, we selected optimal subsets with 5, 10, or 20 features. Models were then retrained, without regularization, using only the feature subset to evaluate its performance. Models were also trained on all 70 features. This procedure was repeated for 30-fs time windows spanning -200 fs to 0 fs to evaluate the predictive performance of LR models over time.

NN models were developed using the multilayer perceptron classifier from the scikit-learn module in Python. All NNs were constructed with one hidden layer with 70 nodes and ReLU activation (this architecture consistently had the most favorable Bayesian information criterion (BIC)[21,22] scores, and we saw limited predictive performance improvement when increasing the number of hidden layers or nodes), and trained using a learning rate of 0.001, $\alpha = 0.0001$, and batch size of 200. These choices were supported by a grid search over hyperparameter values. To evaluate NN performance when using feature subsets, greedy sequential feature selection was used to choose high-performing subsets with only 5, 10, or 20 features, on which NNs were retrained and evaluated. Models were also trained on all 70

features. This procedure was repeated for 30-fs time windows spanning -200 fs to 0 fs to evaluate the predictive performance of NN models over time.

We report all models' AUROC (area under the receiver operating characteristic curve) and accuracy on held-out data using 5-fold cross validation. In every fold, each trajectory was used in either training or testing but not both. That is, the training and testing sets never included time points from the same trajectory.

Protein Redesign

We implemented a protein redesign procedure to find mutations that energetically stabilized reactive-like conformations relative to non-reactive-like ones, based on WT. Subsets of representative reactive-like and non-reactive-like structures were selected and used to construct the objective function in multistate protein redesign. To select characteristic structures, LR and NN models trained on structures from -160 to -130 fs were used to score all enzyme-substrate complexes that were sampled within that time window (3,287,922 unique structures); here, the score represents the model's estimated probability that a given structure will proceed to successfully react (i.e., is a reactive structure). To choose a LR model to select structures, we sought to use a simple model (few input features) that still achieved reasonable performance (AUROC > 0.70) at early time points, as it was assumed that a simple description of the differences between reactive and non-reactive enzyme-substrate conformations would be easier to design. As such, the 10-feature LR model trained on structures from -160 to -130 fs, which had an AUROC of 0.701 (64.5% accuracy), was used (Table S2). The NN model used for selecting structures was similarly trained on structures from -160 to -130 fs. But, unlike the selected LR model, the NN was allowed to use all 70 features with the goal of prioritizing model performance over parsimony. This model had an AUROC of 0.896 (81.4% accuracy).

In the first design iteration, we chose three reactive structures that were near the upper quartile in LR-assigned reactive structure scores, and we chose three non-reactive structures

that were near the lower quartile in LR-assigned non-reactive structure scores. In making these selections, we avoided choosing multiple structures from the same pathway ensemble. For the second design iteration, we used the same set of non-reactive structures from before, but we updated the selection of the reactive structures. The original set of reactive structures was chosen based on score alone, without regard for how the reactive structures compared to the non-reactive structures. In the second design iteration, we sought to create corresponding pairs of non-reactive and reactive structures by choosing, for each non-reactive structure, an idealized reactive structure that was directly across the LR decision boundary from the non-reactive structure. Specifically, for each non-reactive structure, we defined an ideal point in feature space that lay an equal distance away from the LR decision boundary on the other (reactive) side, such that the line connecting the non-reactive structure to the ideal point was orthogonal to the LR decision boundary. We then selected from among the reactive structures that were closest to this ideal point, as measured by Euclidean distance. The goal of this procedure was to create structure pairs (one reactive and one non-reactive) that were as similar as possible except in features identified by the LR model as crucial in determining reactivity. In design iteration three, new sets of three reactive and three non-reactive structures were selected from among those that were within 1% of the modes of the reactive and non-reactive structures' score distributions, respectively, for NN models. We paired each non-reactive structure with the eligible reactive structure that was nearest it in feature space, measured by Euclidean distance. This same set of structures was used for the fourth design iteration.

OSPREY 3.0[23] was used to implement the COMETS algorithm[24] for multistate DEE/A*-based protein redesign. In brief, the algorithm identified new side chain identities that minimized an energetic design objective that was defined over multiple protein structures. Here, the algorithm treats the minimum energy conformation (subject to constraints) in each protein structure for every putative amino acid sequence.[24] We defined the design objective f, a function of sequence s, as $f(s) = \sum_{i=1}^{N_R} E_{R,i} - \sum_{i=1}^{N_{NR}} E_{NR,i}$ where N_R and N_{NR} are

respectively the numbers of reactive and non-reactive structures (three here), $E_{R,i}$ is the potential energy of the ith reactive structure, and $E_{NR,i}$ is the potential energy of the ith non-reactive structure. The design objective score for each mutant s was reported as the difference between $f(s)$ and the corresponding objective score for the WT sequence, $f(WT)$. The potential energies were calculated using the AMBER force field.[23–25] Parameters for substrate, Mg^{2+} ions, acetylated alanine, and NADPH were added using the antechamber module in AmberTools20.[26] A discrete rotamer library based on that used by Lippow *et al.* was used for all designs,[27] with the water residues, substrate, NADPH, and Mg^{2+} ions held fixed. Solvation penalties were treated using EEF1.[23,25,28]

Two types of designs were conducted: single mutant and double mutant. For single mutant designs, a single site was permitted to mutate to any side chain (different protonation states for histidine were handled as different residues) and conformationally repack. Any other residues with one or more atoms within 5 Å of the mutable site were allowed to repack and adopt a new conformation but were restricted from mutating. In a given single mutant design round, this procedure was repeated for each site among a set of selected mutable sites. For double mutant designs, a subset of mutable sites was selected and the design of double mutants was conducted in two stages. In the first stage, all sites among the mutable set were subjected to standard single mutant design as previously described. In the next stage, all of the generated single mutants were considered for an additional, secondary mutation at each of the other sites in the mutable set. This two-stage design procedure, as opposed to mutating and designing two sites simultaneously, was done to reduce the conformational search space and runtime, though it does compromise on accuracy. For each site design, stability constraints were enforced such that the designed mutations' energetically optimized reactive structures were no more than 5 kcal/mol less stable than the starting sequence's optimized reactive structures, otherwise the mutation was disallowed. Up to five unique sequences were returned by each design provided that each sequence's objective score was within +20 kcal/mol of the best-performing sequence. Mutants that were selected for further

characterization and screening with QM/MM simulations were first equilibrated for 200-ps.

The first three design iterations all generated single mutants. The fourth design iteration generated double mutants.

Mutant Characterization and Screening

Following protein redesign rounds, each mutant was evaluated by its objective score, its dynamics in the reactant well, and in some cases its approximated reaction energy barrier. The objective score was computed as described in Protein Redesign. Reactant well dynamics were tracked by running 30 independent 400-ps QM/MM dynamics simulations in the reactant well, as described in TIS Rate Constant Computations for the determination of the flux factor. The 70 structural features described in Machine Learning were recorded at every 1,000 time steps, and the fraction of time steps that were on the reactive side of the machine learning classifiers was recorded. The objective score, and the fractional population of reactive-like structures in the reactant well, verified that a given mutant more frequently adopted reactive-like structures than WT did, as was the design goal. However, if a given mutant more frequently sampled reactive-like conformations than WT, it could still have had a higher activation energy ($\Delta G^{\ddagger}$), which was a common failure mode in early testing. Therefore, we updated later screening stages by calculating for each mutant an approximate PMF curve along λ as described in Potential of Mean Force Calculations. These PMF curves were used to approximate $\Delta G^{\ddagger}$ for each mutant. We then prioritized candidate mutants for more expensive TIS k_{cat} calculations if their $\Delta G^{\ddagger}$ was lower than, or comparable to, WT's, and we ruled out mutants that were expected to have significantly larger energy barriers than WT.

Tracking Reactive-like Structural Characteristics

Statistics were tracked for three reactive-like structural characteristics that were observed across multiple mutants with significantly increased calculated activity relative to WT: loss

of Q136-NADPH hydrogen bonds, rotated E319 side chains, and eclipsed C_5 conformations. We defined several criteria to track when these characteristics were present. We considered the Q136-NADPH hydrogen bond to be present only if the distance from Q136's donated proton to the amide carbonyl of NADPH (the acceptor) was less than 2.3 Å, the angle between the axis from Q136's amine nitrogen to its donated proton and the purported hydrogen bond was within 30 degrees of 180 degrees, and the angle between the purported hydrogen bond and NADPH's amide carbonyl group was within 30 degrees of 120 degrees. If any of these three conditions was not met, then the conformation was considered to have no Q136-NADPH hydrogen bond. The rotated E319 conformation was recorded as present for conformations in which the angle between the axis spanning E319's carboxylate oxygens and the axis spanning atoms C_4 and O_6 in the substrate was less than 75 degrees. An eclipsed C_5 conformation was reported if the dihedral angle measured between any one of the hydrogens bonded to C_5 and either the bulky O_6 bonded to C_4 or O_8 bonded to C_7 was less than 30 degrees.

Statistics

Statistics were performed using SciPy in Python. Values are reported as the average ± the standard error of the mean (SEM). Normality of data was evaluated using an F-test. Equality of variance was evaluated using the Shapiro-Wilk test. For normal data with equal variance, comparisons between two groups were performed using a t-test. For data that were not normal and/or had unequal variance, comparisons between two groups were performed using a Mann-Whitney U test. Specifically, for the comparison of mutant and WT k_{cat} values calculated with TIS, a one-sided Mann-Whitney U test was used (the alternative hypothesis being that the mutant value was larger than the WT one), and significance was detected using the Benjamini-Hochberg procedure to control the false discovery rate (FDR) at $\alpha = 0.05$.

References

(1) Biou, V.; Dumas, R.; Cohen-Addad, C.; Douce, R.; Job, D.; Pebay-Peyroula, E. The crystal structure of plant acetohydroxy acid isomeroreductase complexed with NADPH, two magnesium ions and a herbicidal transition state analog determined at 1.65 Å resolution. *EMBO J.* **1997**, *16*, 3405–3415.

(2) Berman, H. M.; Westbrook, J.; Feng, Z.; Gilliland, G.; Bhat, T. N.; Weissig, H.; Shindyalov, I. N.; Bourne, P. E. The Protein Data Bank. *Nucleic Acids Res.* **2000**, *28*, 235–242.

(3) Bonk, B. M.; Weis, J. W.; Tidor, B. Machine Learning Identifies Chemical Characteristics That Promote Enzyme Catalysis. *J. Am. Chem. Soc.* **2019**, *141*, 4108–4118.

(4) Silver, N. W. Ensemble methods in computational protein and ligand design : applications to the Fc[gamma] immunoglobulin, HIV-1 protease, and ketol-acid reductoisomerase systems. Ph.D. Dissertation, Massachusetts Institute of Technology, **2012**.

(5) Brooks, B. R.; Bruccoleri, R. E.; Olafson, B. D.; States, D. J.; Swaminathan, S.; Karplus, M. CHARMM: A program for macromolecular energy, minimization, and dynamics calculations. *J. Comput. Chem.* **1983**, *4*, 187–217.

(6) Brooks, B. R. et al. CHARMM: the biomolecular simulation program. *J. Comput. Chem.* **2009**, *30*, 1545–1614.

(7) Proust-De Martin, F.; Dumas, R.; Field, M. J. A hybrid-potential free-energy study of the isomerization step of the acetohydroxy acid isomeroreductase reaction. *J. Am. Chem. Soc.* **2000**, *122*, 7688–7697.

(8) Frisch, M. J. et al. Gaussian 03, Revision C.02. Gaussian, Inc., Wallingford, CT, 2004.

(9) Seelam, N. Computational approaches to understand the atomistic drivers of enzyme catalysis. Ph.D. Dissertation, Massachusetts Institute of Technology, **2021**.

(10) Dewar, M. J.; Zoebisch, E. G.; Healy, E. F.; Stewart, J. J. Development and use of quantum mechanical molecular models. 76. AM1: a new general purpose quantum mechanical molecular model. *J. Am. Chem. Soc.* **1985**, *107*, 3902–3909.

(11) Stewart, J. J. Optimization of parameters for semiempirical methods IV: Extension of MNDO, AM1 and PM3 to more main group elements. *J. Mol. Model.* **2004**, *10*, 155–164.

(12) Huang, J.; Mackerell, A. D. CHARMM36 all-atom additive protein force field: Validation based on comparison to NMR data. *J. Comput. Chem.* **2013**, *34*, 2135–2145.

(13) Gao, J.; Amara, P.; Alhambra, C.; Field, M. J. A generalized hybrid orbital (GHO) method for the treatment of boundary atoms in combined QM/MM calculations. *J. Phys. Chem. A* **1998**, *102*, 4714–4721.

(14) Kumar, S.; Rosenberg, J. M.; Bouzida, D.; Swendsen, R. H.; Kollman, P. A. The weighted histogram analysis method for free-energy calculations on biomolecules. I. The method. *J. Comput. Chem.* **1992**, *13*, 1011–1021.

(15) Grossfield, Alan WHAM: the weighted histogram analysis method. `http://membrane.urmc.rochester.edu/wordpress/?page_id=126`, Accessed: 2019-10-15.

(16) Van Erp, T. S.; Moroni, D.; Bolhuis, P. G. A novel path sampling method for the calculation of rate constants. *J. Chem. Phys.* **2003**, *118*, 7762–7774.

(17) Dellago, C.; Bolhuis, P. G.; Geissler, P. L. *Advances in Chemical Physics*; John Wiley & Sons, Inc., 2002; Chapter 1, pp 1–78.

(18) Geissler, P. L.; Chandler, D. Importance sampling and theory of nonequilibrium solvation dynamics in water. *J. Chem. Phys.* **2000**, *113*, 9759–9765.

(19) Geissler, P. L.; Dellago, C.; Chandler, D. Chemical dynamics of the protonated water

trimer analyzed by transition path sampling. *Phys. Chem. Chem. Phys.* **1999**, *1*, 1317–1322.

(20) Tibshirani, R. Regression Shrinkage and Selection Via the Lasso. *Journal of the Royal Statistical Society: Series B (Methodological)* **1996**, *58*, 267–288.

(21) Schwarz, G. Estimating the Dimension of a Model. *The Annals of Statistics* **1978**, *6*, 461–464.

(22) Kass, R. E.; Wasserman, L. A Reference Bayesian Test for Nested Hypotheses and its Relationship to the Schwarz Criterion. *J. Am. Stat. Assoc.* **1995**, *90*, 928–934.

(23) Hallen, M. A.; Martin, J. W.; Ojewole, A.; Jou, J. D.; Lowegard, A. U.; Frenkel, M. S.; Gainza, P.; Nisonoff, H. M.; Mukund, A.; Wang, S.; Holt, G. T.; Zhou, D.; Dowd, E.; Donald, B. R. OSPREY 3.0: Open-source protein redesign for you, with powerful new features. *J. Comput. Chem.* **2018**, *39*, 2494–2507.

(24) Hallen, M. A.; Donald, B. R. comets (Constrained Optimization of Multistate Energies by Tree Search): A Provable and Efficient Protein Design Algorithm to Optimize Binding Affinity and Specificity. *J. Comput. Biol.* **2016**, *23*, 311–321.

(25) Gainza, P.; Roberts, K. E.; Georgiev, I.; Lilien, R. H.; Keedy, D. A.; Chen, C.-Y.; Reza, F.; Anderson, A. C.; Richardson, D. C.; Richardson, J. S.; Donald, B. R. In *Methods in Protein Design*; Keating, A. E., Ed.; Methods in Enzymology; Academic Press, 2013; Vol. 523; pp 87–107.

(26) D.A. Case *et al.* AMBER 2020. University of California, San Francisco.

(27) Lippow, S. M.; Tidor, B. Progress in computational protein design. *Curr. Opin. Biotechnol.* **2007**, *18*, 305–311.

(28) Lazaridis, T.; Karplus, M. Discrimination of the native from misfolded protein models

with an energy function including implicit solvation 11Edited by A. R. Fersht. *J. Mol. Biol.* **1999**, *288*, 477–487.
